# Adherence to anti-seizure medications among persons living with epilepsy attending a community- based epilepsy clinic in Buikwe and Mukono Districts-Uganda: A mixed methods study

**DOI:** 10.1371/journal.pone.0356494

**Published:** 2026-08-21

**Authors:** Ronald Kibirige, Marianna Agaba Nyangire, Mark Kaddumukasa, Christine Muhumuza, Christopher Burant, Shirley Moore, Elly T. Katabira, Martha Sajatovic, Jane Tugume, John Nsubuga, Nazarius Mbonna Tumwesigye, Aggrey David Mukose

**Affiliations:** 1 Department of Epidemiology and Biostatistics, School of Public Health, College of Health Sciences, Makerere University, Kampala, Uganda; 2 Department of Disease Control and Prevention, School of public Health, College of Health Sciences, Makerere University, Kampala, Uganda; 3 Department of Medicine, School of Medicine, College of Health Sciences, Makerere University, Kampala, Uganda; 4 Louis Stokes VA Medical Center, Geriatric Research Education, and Clinical Center, Cleveland, Ohio, United States of America; 5 Frances Payne Bolton School of Nursing, Case Western Reserve University, Cleveland, Ohio, United States of America; 6 Neurological and Behavioral Outcomes Center, University Hospitals Cleveland Medical Center & Case Western Reserve University School of Medicine, Cleveland, Ohio, United States of America; Niigata University of Health and Welfare: Niigata Iryo Fukushi Daigaku, JAPAN

## Abstract

**Background:**

Epilepsy is a common neurological disease especially in Sub-Saharan Africa. Medication adherence is essential in seizure control; however, majority of patients living with epilepsy have poor adherence to anti- seizure medications. The purpose of this study was to determine the prevalence, the factors associated with, and the experiences with adherence to anti-seizure medications among people living with epilepsy attending a community- based epilepsy clinic in Buikwe District and Mukono General Hospital in Mukono District, central-Uganda.

**Methods and material:**

In this cross-sectional mixed methods study, 277 participants were enrolled and 20 in-depth interviews were conducted from April to July 2024. Socio-demographics and treatment related information were collected using structured questionnaires. Medication adherence was measured using the standardized 8- item Morisky Medication Adherence Scale. Modified Poisson regression was done to determine factors associated with adherence to anti-seizure medications. Thematic analysis was conducted to explore participant’s experiences with adherence to anti-seizure medications.

**Results:**

The overall prevalence of adherence to anti-seizure medications was 66.5%. Factors significantly associated with adherence to Anti-seizure medications were earning a modest monthly income, adjusted prevalence ratios [aPR], (aPR 1.29; CI 1.13-1.53), receiving an inadequate treatment dosage (aPR 0.80; 95% CI 0.64-0.92), waiting for three or more hours at the facility (aPR 0.79: 95% CI 0.61-0.91), and being aged 30 to 34 years (aPR 0.71: CI 0.53-0.95). Key themes describing experiences with adherence to anti-seizure medications included fear of stigmatization, fear of injury and loss of life, social support, financial burden, individual motivation, misconceptions about anti-seizure medications, and limited awareness about their effectiveness.

**Conclusion:**

This study revealed that about two- thirds of people living with epilepsy were adherent to anti-seizure medications. There is a need to implement strategies such as incorporating a medication adherence tool into routine patient care to improve adherence in our settings.

## Introduction

Epilepsy is a chronic neurological disorder affecting approximately 50 million people globally. It is estimated that 80% of people living with epilepsy (PLWE) reside in developing countries like Uganda [1,2]. The crude prevalence of epilepsy in Uganda is reported to be 16.9 per 1000 persons [3]. The risk of premature death due to epilepsy is 3 times higher than for the general population, especially among those not receiving regular anti-seizure medications [4]. Anti-seizure medications (ASMs) remain a mainstay of epilepsy treatment with about 70% of PLWE becoming seizure free with ASM treatment [5]. Nearly 90% of PLWE in low and middle- income countries are not receiving appropriate treatment, which could be due to low availability of ASMs, misconceptions about ASMs, perceived epilepsy stigma and cultural attitudes. Thesemay lower the patient’s level of adherence to treatment [6]. Adherence is the most important modifiable factor that can improve treatment outcomes and reduce mortality [7].

Patients who are non-adherent to their medication are frequently hospitalized, often with prolonged lengths of hospital stay [8].

Approximately 25% of prescribed medications are omitted by patients, which might lead to morbidity and mortality including increased hospitalization, unnecessary and costly procedures and treatment failure [9].

The explanatory factors for the low adherence levels are centered around low public awareness, limited diagnostic and treatment resources, beliefs about the treatment and availability of highly trusted non allopathic treatments [10,11]. The risk of experiencing successive seizures increases by 21% among patients who do not adhere to their prescribed treatment [12]. Studies in Uganda show that poor adherence to epilepsy treatment is related to social stigma and misconceptions about the condition [13]. Therefore, we set out to determine the level of adherence, the factors associated with adherence, and experiences with adhering to anti-seizure medications among persons living with epilepsy attending a community -based epilepsy clinic in Buikwe District or Mukono General Hospital, Mukono District. Facility records show increasing numbers of people with epilepsy seeking care at both the community-based epilepsy clinic in Buikwe and at Mukono General Hospital.

## Methods

### Study settings

This study was carried out in a community-based epilepsy clinic in Buikwe District and at Mukono General Hospital, Mukono District. The community- based epilepsy clinic is in central Uganda, about 60kms from Kampala, the capital city of Uganda. The clinic is carried out monthly by Butabika National Referral Hospital, community epilepsy program in collaboration with the Little Sisters of St Francis. Facility records indicate that the number of people living with epilepsy (PLWE) attending this clinic daily is increasing, with attendance often reaching 200 or more patients per day.

Mukono General Hospital is in Mukono town, about 35kms west of the headquarters of Buikwe. More than 100 people living with epilepsy attend a weekly epilepsy clinic at Mukono General Hospital, a government facilitated hospital that provides free anti-seizure medications. The most prescribed anti-seizure medications at both the community-based epilepsy clinic and Mukono General Hospital are; Phenytoin, Carbamazepine, Phenobarbitone, Sodium valproate and Lamotrigine

We pooled all these participants from the two clinics, i.e., the community-based epilepsy clinic, and Mukono General Hospital into a single cohort since they serve as epilepsy treatment centers within the same geographical area, with similar clinical follow-up procedures, medication regimens, and patient counseling practices. In addition, these two sites draw patients from overlapping communities within Buikwe, Mukono and surrounding areas, resulting in similar socio-demographic and health system contexts for patients attending either facility. The baseline characteristics of participants from both sites were also assessed and showed no meaningful differences in key variables such as age distribution, sex, socioeconomic indicators, and duration on treatment. Furthermore, a site-level comparison of key outcomes also revealed no significant variation in adherence patterns between the two facilities.

### Study design and population

The study was a cross-sectional design conducted both in the community and at the hospital, employing quantitative and qualitative methods [14]. The study adhered to the Strengthening the Reporting of Observational Studies in Epidemiology (STROBE) guidelines [15]. The study population consisted of persons living with epilepsy15 years and above attending regularly at the community-based epilepsy clinic in Buikwe District, and Mukono General Hospital, Mukono District. The study was conducted from 05^th^ April to 20^th^ July 2024. Participants were approached through mental health care workers, and community health workers, who introduced the research team to the study respondents.

### Sample size calculation

For the quantitative component, a sample size of 277 was determined using the modified Kish-Leslie formula for cross-sectional studies, (1965) [16].


$$\begin{array}{c} \text{N }={\text{Z}}^{\text{2}}\text{P }(\text{1}-\text{ P})/{\text{d}}^{\text{2}} \end{array}$$

where N represents the sample size; d is the margin of error set at 5%; Z is the standard normal deviation corresponding to a 95% confidence level (1.96); and P is the assumed proportion of patients adhering to anti-seizure medications (P = 0.209), derived from studies conducted in Iganga and Mayuge Districts [17].


$$\begin{array}{c} \begin{array}{c} \text{N}={\text{1}.\text{9}\text{6}}^{\text{2}}\times \text{0}.\text{2}\text{0}\text{9}(\text{1}-\text{0}.\text{2}\text{0}\text{9})/{\text{0}.\text{0}\text{5}}^{\text{2}}\\ \text{N }=\text{ 254} \end{array} \end{array}$$

To account for potential non response, an additional 10% of respondents were included [18].


$$\begin{array}{c} \begin{array}{c} \text{N }=(\text{254 }\times \text{0}.\text{1})+\text{ 254}\\ \text{N }=\text{ 279} \end{array} \end{array}$$

During data collection, 2 respondents who were; a male and a female aged 30 and 50 years, respectively from urban areas, and attending to Mukono General Hospital mental health clinic declined participation in the study. Therefore, 277 study participants were enrolled for the quantitative component of the study.

A proportionate distribution of 1:2 was considered, whereby 184 and 93 patients were selected from the community- based epilepsy clinic in Buikwe and Mukono General Hospital, respectively. Based on the hospital records, approximately 200 patients aged 15 years and above receive treatment at a community-based epilepsy clinic in Buikwe, while about 100 patients of the same age group attend the epilepsy clinic at Mukono General Hospital

For qualitative research sub study, 20 respondents who were purposively selected based on the number of years they had been using anti-seizure medications, and this was considered important for capturing a range of experiences related to treatment adherence. This sample size is within the recommended number of 20–50 individuals [19,20]

### Inclusion and exclusion criteria

Participants who met the following inclusion criteria: a) had a confirmed diagnosis of epilepsy, were regularly attending the clinics, and had been receiving anti- seizure medications for the last 6 months; b) were aged 15 years and above and; c) provided written informed consent or assent were included in the study. We excluded patients who were severely ill, requiring admission or who had cognitive impairment that limited their ability to actively participate in the study activities.

### Sampling procedure

Consecutive sampling was employed to select the study participant for the quantitative part of the study, until the required sample size of 277 respondents was attained.

In the qualitative part of the study, respondents were stratified by age, sex and level of education. The strata according to age in years were 15–18, 19–30, 31–40, 41–50 and >50. For the sample size of 20, saturation was reached when no new information emerged from the interviews.

### Data collection

Data were collected using a structured questionnaire and face‒to-face in-depth interviews were conducted for the qualitative part. Data on prevalence of adherence to anti-seizure medications was determined using the 8- item Morisky Medication Adherence Scale (MMAS-8) [21]. The questions asked included; whether the patient forgot to take the medications the previous day, ever felt hassled about taking medication, and stopping to take medication when symptoms reduce. This scale consists of 8 items; each of the first 7 items has 2 possible responses (yes/no), while the 8th item is answered with a 5-point Likert scale. The possible total medication adherence score ranges between 0 and 8, with a higher score indicating good adherence. A total score < 6 isconsidered low adherence, while ≥ 6 but < 8 indicates moderate adherence, and a score of 8 indicates high adherence [22]. Adherence to ASMs being a binary outcome for this study, scores of < 6 were categorized as low adherence, while scores of 6 to 8 adherence.The scale has a sensitivity of 93%, and a specificity of 53% on measuring ASMs adherence in similar settings [23].

Two translators independently translated the English version of the MMAS-8 to Luganda, the dominant widely used language in Buikwe and Mukono Districts, Uganda. A third translator performed reverse translation from Luganda to English. The original and back translated versions were compared and inconsistencies were resolved. A pilot test was performed in 10 patients living with epilepsy and the interpretation of the tool was consistent. The patients who participated in this face- validity did not participate in this study.

Regarding factors associated with adherence to anti-seizure medications, this study adopted the World Health Organization Multidimensional Adherence Model (WHO-MAM) [24].This ecological model combines intrapersonal, interpersonal, organizational, policy, and community factors, to understand adherence behavior [25]. This WHO-MAM categorizes factors associated with adherence to ASMs in five groups: socioeconomic factors, health system-related factors, patient-related factors, condition-related factors and treatment-related factors. The study participants were asked about their socioeconomic characteristics such as age, sex, area of residence, monthly income and level of education, age was recorded in complete years. The participants also provided information on patient-related factors such as family support; including whether a family member accompanies them to the facility and whether a family member encourages them to take the medications. Additional information was collected on their independence in activities of daily living, the number of clinic visits made in the previous six months, and their self perceived quality of life. Participants also provided information on condition-related factors, including alcohol use, the number of anti-seizure medications taken per day, the number of epileptic attacks they experienced per week, and presence of other chronic conditions. In addition, data were collected on health system-related factors such whether they receive follow-up care at home by the health care providers, whether they receive an adequate treatment dosage whenever they go to the facility, the average waiting time before receiving medication, the quality of patient-provider communication level, and the availability of medications whenever they go to the facility. Finally, participants were asked to provide information on therapy-related factors, including duration of time they had been using their medication, whether their medications had been changed in the previous three months, whether they experienced any side effects when taking the medication, and whether they perceived any benefits from their treatment. They were also asked about the availability of a health worker within 5kms to manage any complications arising from medication use, as well as the presence of a referral system in case further consultation was needed. Those questionnaires were interviewer-administered to the enrolled patients in Luganda. As part of the study’s quality control measures, two research assistants (a community-based health worker and a psychiatric nurse) were trained for two days on the objectives, key methods of the study, how to fill the tools, consent process and how to collect quality data. All the research assistants were adequately and regularly supervised by the Principal investigator and the co-investigator. The supervision entailed checking on the completeness and accuracy of the completed questionnaire from the previous interview. The research assistants were availed for each clinic day. The tool for capturing data on factors associated with adherence to ASMs was also pretested among 10 patients living with epilepsy attending a community-based epilepsy clinic in Buikwe District and Mukono General Hospital who consented to participate in this piloting. The purpose of this piloting was to check for its feasibility in terms of language suitability, potential to eliminate bias and consistence. The patients who participated in piloting did not participate in this study.

Regarding experiences with adherence to anti-seizure medications, this study used face-to-face in-depth interviews (IDIs) with an unstructured interview guide to gather participants’ information. As part of the study’s quality control measures, the interview guide was pretested among 5 patients attending a community-based epilepsy clinic, and Mukono General Hospital who consented to take part in the pilot study. An experienced qualitative researcher was present and assisted in taking note of the non-verbal cues. Each interview lasted between 30 minutes to 1hour. All patients were interviewed in Luganda. For English words that had no direct translation to Luganda, consensus was reached and suitable ways of conveying them to participants agreed.

### Data analysis

#### Quantitative data.

Data were sorted, edited and entered into excel sheet. Data cleaning was then done to ensure consistence and completeness and then imported into statistical software STATA version 14. We summarized continuous variables using the means and medians and categorical variables summarized by proportions. Modified Poisson regression was used at bi-variable and multivariable analysis to identify factors associated with adherence to ASMs. Based on the outcome variable for this study (binary outcome), logistic regression would be the appropriate regression to use, however the prevalence of adherence to anti-seizure medications used in this study was > 10%, rendering the regression model for use to change to modified Poisson because the use of logistic regression would under-estimate the strength of association [26].The modified Poisson regression model is the Poisson regression of binomial data using robust error variance [27].We conducted backward and forward methods of model building to eliminate and add variables one at a time at each level of model building. This method is used to determine the form of the variables that yield the best model fit (lowest AIC). A p-value threshold of 0.05 was used to assess statistically significant associations. Adjusted prevalence ratios, 95% confidence intervals and corresponding p-values were recorded. All analysis was done using STATA version 14.

#### Qualitative data analysis.

Qualitative data were concurrently transcribed verbatim. Transcripts were coded independently by the analysts (BT, JN, and TJ). Transcriptions and translations were checked by these three analysts against the recordings for correctness of information before proceeding to the next set of interviews. These analysts then reconciled the suggested codes which were merged into themes and subthemes. Thematic analysis was done using Atlas Ti version 8, following the procedures recommended by Miles & Huberman [28]. We followed an iterative process of data analysis [28]. We followed the four major steps in data analysis [29]: 1) Data familiarization: the transcripts were read multiple times by the three analysts, 2) Development of a coding framework: a descriptive coding scheme was inductively generated by the analysts. 3) Abstraction of coded data into thematic categories: the emergent codes were then abstracted into thematic matrices [30] using Atlas ti software and 4) Interpretation and overall synthesis by the PI and the analysts following multiple reading of the transcripts [30]. Results were presented according to themes and subthemes with associated quotations from the study respondents. Use of thematic analysis was based on the rationale that it can easily identify, analyze, organize, describe and report themes available in the data [31]. The themes were linked together by the thematic inductive process which involves coding the data without considering the preconceptions of the analysts [32].

### Ethical consideration

We obtained approval to carry out the study from Makerere University School of Public Health Research and Ethics Committee (approval number MakSPH-REC 351).The study was conducted in accordance with the ethical guidelines of the 1975 Declaration of Helsinki and principles of good clinical practice [33]. Administrative permission was obtained from the respective clinics. Written informed consent was obtained from the participants before enrollment into data collection. Assent for those aged less than 18 years was obtained. Confidentiality was maintained by using unique identification numbers instead of names of participants.

### Inclusivity in global research

Additional information regarding the ethical, cultural, and scientific considerations specific to inclusivity in global research is included in the Supporting Information (S1 Checklist)

## Results

### Characteristics of the study respondents

Table 1 summarizes the socio-demographic characteristics of study participants. A total of 277 people living with epilepsy who were available during the study period were included in this study. The mean (SD) age of the respondents was 30.6 ± 13.7 years, and more than a half 52.0% were male, and over (63.5%) were not married. Regarding area of residence, majority of the respondents (77.6%) were residing in rural areas. In addition, nearly half (44.8%) had attained primary as the highest level of education. More than half (54.9%) were not involved in any income-generating activity. This study also found that a large proportion of the respondents (32.2%) were Pentecostal. Furthermore, most participants (69.7%) had a monthly income of less than 28 US dollars.

**Table 1:** Socio-demographic characteristics of 277 people living with epilepsy.

Variable	Category	Frequency	Percent
Sex	Male	144	52.0
	Female	133	48.0
Age	Mean plus standard deviation	30.6 ± 13.7	
Marital status	Married	65	23.5
	Divorced	31	11.2
	Single	176	63.5
	Widowed	5	1.8
Place of residence	Rural	215	77.6
	Urban	62	22.4
Highest education level	None	40	14.4
	Primary	124	44.8
	Post primary	113	40.8
Occupation	Farming	47	17.0
	Casual	40	14.4
	Business	34	12.3
	None	152	54.9
	Salaried workers	4	1.4
Religion	Anglican	59	21.3
	Catholic	89	32.1
	Muslim	20	7.2
	Seventh day	13	4.7
	Pentecost	92	33.2
	Others	4	1.5
Monthly income	<28	193	69.7
	≥28	84	30.3

Table 2 summarizes the scores from the Morisky Medication Adherence Scale. Based on the scores from the Morisky Medication Adherence Scale, 184(66.5%) of the respondents were adherent while 93 (33.5%) were low adherent.

**Table 2:** Adherence scores using the Morisky medication adherence scale (N = 277). Prevalence of adherence to anti-seizure medications using the Morisky Medication Adherence Scale (MMAS-8).

Number	Question	Response	Frequency	Percent	Score
**1**	Do you sometimes forget to take your ASMs daily	No	148	53.4	Adherence
		Yes	129	46.6	Low adherence
**2**	Thinking over the past two weeks where there days you forgot to take your ASMs	No	189	68.2	Adherence
		Yes	88	31.8	Low adherence
**3**	Have you ever stopped to take ASMs when not told by the health provider because you felt worse when took it	No	198	71.5	Adherence
		Yes	79	28.5	Low adherence
**4**	When you travel, do you sometimes forget to move along with your ASMs	No	204	73.7	Adherence
		Yes	73	26.3	Low adherence
**5**	Did you take your ASMs yesterday	No	119	57.0	Low adherence
		Yes	158	43.0	Adherence
**6**	When you feel that your symptoms are under control, do you stop taking ASMs	No	228	82.3	Adherence
		Yes	49	17.7	Low adherence
**7**	Have you ever felt hassled about sticking to your ASMs	No	245	88.5	Adherence
		Yes	32	11.5	Low adherence
**8**	How often do you have difficulty remembering to take all your ASMs	No	174	62.8	Adherence
		Yes	103	37.2	Low adherence

**Total scores** 2216.

**Adherence** (1473 ÷ 2216 × 100) = 66.5%.

**Low adherence** (743 ÷ 2216 × 100) = 33.5%.

Out of the 277 respondents, 184 respondents (0.665 × 277 = 184) were adherent, while 93 respondents (0.335 × 277 = 93) were low adherent.

**Calculating the Chronbach’s alpha, using the formula described by Alfin** [34,35].

α=(K)/(K−1)×(Sy2−Si2)/ Sy2)

Where;

α = Chronbach’s alpha.

K = number of items in the scale.

Si = sum of item scores for each item (adherence scores).

S = sum of total scores for all the items.

$\begin{array}{c} \alpha =(\text{8}/\text{8}-\text{1})\times ({\text{2216}}^{\text{2}}-{\text{1473}}^{\text{2}})/{\text{2216}}^{\text{2}}\\ \alpha =\text{0}.\text{64} \end{array}$

### Factors associated with adherence to anti-seizure medications

Table 3 summarizes factors associated with adherence to anti-seizure medications. At bi-variable analysis, there was statistically significant association between the following variables: age of a patient, monthly income, waiting time, receiving medications for other chronic conditions, adequacy of treatment dosage, smoking status, availability of a referral system, the number of times the patient had attended the clinic in the previous six months and dependence in activities of daily living. After adjusting for confounding factors in the multivariable analysis, being aged 30–34 years, earning $28 or more, waiting for three or more hours before receiving medications and receiving inadequate dosage of medications emerged as factors statistically significant on adherence to anti-seizure medications. The prevalence of adherence to anti-seizure medication was 29% lower among patients aged 30–34 years compared to those 15–19 years (adjusted prevalence ratio) [aPR] (aPR 0.71, 95% CI: 0.53–0.95). The prevalence of adherence was 29% higher among patients earning USD 28 or more per month compared to those earning less than $28 (aPR1.29, 95% CI:1.13–1.53). Religious affiliation was not associated with anti-seizure medication adherence.

**Table 3:** Factors associated with adherence to anti-seizure medications.

Variable	Category	Low adherence	Adherence	unadjusted prevalence Ratio (uPR) 95%CI	adjusted Prevalence Ratio (aPR) 95% CI
Age group	15-19	20(30.8)	45(69.8)	1	1
	20-24	14 (27.6)	37(72.5)	0.84(0.65-1.09)	0.90(0.70-1.17)
	25-29	16 (44.4)	20(55.6)	0.70(0.05-1.07)	0.76(0.55-1.07)
	30-34	20 (45.6)	24 (54.6)	0.61(0.43-0.84)*	0.71(0.53-0.95)**
	35-39	6 (31.6)	13 (68.4)	0.94(0.66-1.33)	0.91(0.66-1.24)
	40-44	10 (41.7)	14 (58.3)	0.72(0.50-1.04)	0.69(0.47-1.03)
	≥45	5 (13.2	33 (86.8)	0.86(0.66-1.12)	1.03(0.83-1.29)
Monthly income ($)	< 28	76(39.4)	117 (60.6)	1	1
	≥ 28	16(17.9)	68(82.1)	1.33(1.14-1.56)*	1.31(1.12-1.54)**
Waiting time (hours)	< 3	46 (25.4)	135 (74.6)	1	1
	≥ 3	45 (46.9)	51 (53.1)	0.77(0.62-0.94)*	0.79(0.61-0.91)**
Receiving medications for other chronic conditions	No	38(25.9)	109(74.1)	1	
	Yes	54(41.5)	76(58.5)	0.79(0.66-0.94)*	
Adequacy of treatment dosage	Adequate	36(25.7)	104(74.3)	1	1
	Inadequate	56(40.9)	81 (59.1)	0.79(0.67-0.94)*	0.80(0.64-0.92)**
Smoking status	No	85(36.2)	150(63.8)	1	
	Yes	7(16.7)	35(83.3)	1.31(1.1-1.54)*	
Availability of a referral system	No	79(36.7)	136(63.3)	1	
	Yes	13(21.0)	49(79.0)	1.24(1.06-1.47)*	
Days attended the clinic in the previous 6 months	Once	9(60.0)	6(40.0)	1	
	Twice	13(26.5)	35(81.4)	2.07(1.09-3.91)*	
	Thrice	59(36.7)	102(63.3)	1.86(0.98-3.53)	
	≥Four times	16(27.1)	43(72.9)	1.58(0.82.2.98)	
Dependence in activities of daily living	Independent	29(34.9)	54(65.1)	1	
	Moderately independent	8(47.1)	9(52.9)	0.83(0.53-1.29)	
	Dependent	21(28.4)	53(71.6)	1.04(0.84-1.29)	
	Slightly dependent	7(17.5)	33(82.5)	1.26(1.02-1.55)*	
	Totally dependent	27(42.9)	36(67.2)	0.87(0.67-1.13)	

*candidate variables for multivariable analysis at P-value < 0.05.

** Significant at p-value < 0.05: [1] reference.

The study also showed that the prevalence of adherence was 21% lower among patients who waited three or more hours compared to those who waited three or less hours (aPR 0.79, 95% CI: 0.61–0.91). Additionally, the prevalence of adherence was 20% lower among patients that perceived their treatment dosage as inadequate compared to those who felt they received an adequate dosage (aPR 0.80, 95% CI: 0.64–0.92).

### Experiences of adhering to anti-seizure medications

#### Characteristics of respondents for the qualitative part of the study.

Table 4 summarizes characteristics of 20 respondents interviewed using In-depth interviews. More than half of the respondents were male [12]. Their average age was 30.6years.The majority were single [14] and lived in rural areas [15]. About half were Pentecostals [8], most had attained primary as the highest level of education [13], and nearly half were not engaged in income-generating activities [11].

**Table 4:** Characteristics of respondents for the in- depth interviews N = 20.

Variable	Category	Frequency	Percent
Sex	Male	12	60.0
	Female	8	40.0
Age	Mean age (SD)	30.6 ± 13.7	
Marital status	Married	4	20.0
	Divorced	1	5.0
	Single	14	70.0
	Widowed	1	5.0
Place of residence	Urban	15	75.0
	Rural	5	25.0
Religion	Anglican	4	20.0
	Catholic	5	25.0
	Muslim	2	10.0
	Pentecost	8	40.0
	Other	1	5.0
Highest education level	None	1	5.0
	Primary	13	65.0
	Post primary	6	30.0
Occupation	Farming	4	20.0
	Casual	4	5.0
	Business	11	20.0
	None		55.0

### Themes that emerged for the study

Participants described a wide range of experiences related to adherence to anti-seizure medications; however, for purposes of this study, five themes emerged during the analysis. These were: 1.) Fear of the poor outcomes of epilepsy, 2.) Social support, 3.) Financial burden, 4.) Lack of knowledge on ASMs, misconception about ASMs and community beliefs in traditional powers,and 5.) Individual motivation, including fear of side effects and concerns about taking many medications. The results were presented by themes, each followed by relevant subthemes and supporting quotations from the respondents in italics.

#### Fear of the poor outcomes of epilepsy.

Under the theme of fear of the poor outcomes of epilepsy, two subthemes emerged: Fear of stigmatization and fear of injury and loss of life.

**Fear of stigmatization**

Fear of stigmatization was commonly reported by people living with epilepsy as influencing their adherence to ASMs. Many took their medication as prescribed to avoid episodes that had previously led to stigma, especially in school settings. Participants noted that seizures often resulted in classmates avoiding them due to the misconception that epilepsy is contagious, contributing many leaving schools at the primary level.

*“….When I had missed my medication, I had an epileptic attack when in class. Everyone, including the teacher, ran out. When I was awake, nobody was in the room and everybody was seeing me from the windows, I stopped going to school.”…* (Participant 2, female, 28 years)

Most IDI participants reported that having an epileptic attack in a social gathering caused shame and led them to withdraw from others, affecting their career goals. This fear of experiencing seizures in public motivated many people living with epilepsy to adhere closely to their ASMs

*“…For me, if I miss my medication, I am likely to have an epileptic attack in public, and people will get to know that I have epilepsy. Therefore, I must take my medication on time.”…* (Participant 1, male, 42 years)

**Fear of injury and loss of life**

People living with epilepsy reported that failing to take their medication as prescribed increases the risk of frequent seizures, injuries, and even death. They noted that missing doses often lead to dizziness, fatigue, and repeated falls, which can worsen disabilities and threaten their lives.

“…. *I had not taken my medication that day. When I climbed a jack fruit tree to look for what to eat, I felt weak, dizzy and fell down, only to realize that I had broken my hand and now have a disability. I now must take my medication well to avoid further falls.”…* (Participant 5, male, 37 years)

#### Social support.

Social support including family and community support was frequently mentioned by most PLWE as key factors in their adherence to ASMs.

**Family support**

Family support from immediate caregivers, such as parents, siblings, and grandparents, was mentioned by most participants as crucial for adherence to ASMs. Caregivers, peers, and fellow patients reminded them to collect and take their medication, sometimes picking up medications on their behalf or ensuring they carry them when traveling. Participants also reported receiving priority meals and being protected from risky domestic tasks like fetching water, digging, and cooking, all of which encouraged consistent medication adherence.

*“….When I was cooking food for my young siblings, I got an epileptic attack, fell and burned my leg in fire, the leg has failed to heal. My father stopped me from cooking and asked everyone at home to always cook and give me food on time, such that the medications can work for me.”*(Participant 4, female, 27 years)

**Community support**

Community support for people living with epilepsy (PLWE) included initiatives to help them adhere to their medications. Some community members provide money or reduced transport fees to ensure patients attend clinic appointments, while the community health worker could collect medications on their behalf. Additionally, community members remind PLWE to take their medications, assist with household tasks, provide food, and help keep them safe during epileptic attacks.

*“…If my people are in the garden, the neighbors always give me food, such that I can take my medicines on time.”….* (Participant 17, female, 27 years)

#### Financial burden.

Financial burden emerged as a theme on experiences to low adherence to ASMs, with cost of transport and cost of medications being subthemes.

**High costs of transport**

Transport costs, including motorcycle and car fees, affected majority of the PLWE living five or more kilometers from epilepsy clinics. Many patients discussed that they could not afford these costs and had to walk long distances, which was reported to worsen their seizures and contribute to low adherence to ASMs.

**High cost of anti-seizure medications**

Cost of ASMs included expenses incurred by PLWE when purchasing medications unavailable at the facilities.

Most PLWE reported that ASMs are expensive when they are unavailable, often preventing patients from buying them and making it difficult to follow healthcare providers’ instructions.

“…*Because of the costs, I do not always pick my medication, which makes it difficult for me to take them as instructed by the health care providers, so I did not go to pick medications last time.”*…. (Participant *3, male, 27 years*)

#### Lack of knowledge on ASMs, misconception about anti-seizure medications and community beliefs in traditional powers.

**Lack of knowledge on ASMs**

We defined lack of knowledge on ASMs based on patients’ perceptions whether ASMs control seizures or not. Most PLWE who had difficulty in using ASMs as instructed by health care providers reported limited understanding of how medicines work, while others believed that these medicines are effective when combined with spiritual support. Many also mentioned that they first sought care from traditional healers and only turned to ASMs after traditional treatments failed to improve their condition.

*“…I have taken these medications as instructed by the health care providers for about 10 years, but I don’t think they work. With all the time I’ve been taking them, I feel I should have been healed by now”* (Participant 6, male, 28 years)

**Misconception about anti-seizure medications**

Misconception referred to patients’ negative beliefs about medications. Many PLWE who struggled to use ASMs as instructed believed that epilepsy cannot be treated with modern medication and that one must first address ancestral issues for the condition to resolve. Some participants also claimed that health workers sometimes give expired medicines, which they felt worsened their health instead of helping.

*“….My friend, I have to tell you that we are trying to use these medications as instructed by health care providers; however, we come for them after those other medications have failed to work.”…*(Participant 4,male 27 years)

**Community beliefs in traditional powers**

Community beliefs in traditional powers were widely reported by most participants as contributing to low adherence to ASMs. Many PLWE said their communities believe that epilepsy results from curses, witchcraft, or ancestral wrongdoing, leading families to discourage the use of ASMs. Some participants reported claims that PLWE come from families affected by incurable conditions such as HIV, making ASMs ineffective. These strong traditional beliefs cause some patients to stop or misuse their medications.

*“….They believe that our family was cursed, even if I take these medications, the curse will not go away unless we change our behaviors.”….* (Participant 3, male 27 years)

#### Individual motivation, fear of side effects and too many or too few anti-seizure medication.

Under this theme most individuals reported that personal motivation encouraged adherence to ASMs, while fear of side effects and high number of prescribed medications contributed to low adherence

**Individual motivation**

Individual motivation referred to the personal drive to take ASMs. Most patients reported that they adhered to their medications to prevent seizures that could interfere with their income-generating activities. However, many who were employed reported losing their jobs once employers learned of their condition. Several participants also noted that people with epilepsy often run businesses but hide their status for fear of losing customers, even sending community health workers to collect their medications to avoid being seen at the clinic. This desire to protect their livelihoods leads some patients to conceal their condition, even when their names appear in clinic records.

*“….When I was taken to Kampala to porter on a building, my boss got to know that I get epileptic seizures, they asked me to go back home, I now have to ensure that I take my medications not to get epileptic seizures.”…* (Patient 13, male 26 years)

**Fear of side effects**

Most IDI participants reported that ASMs made them feel dizzy, weak, excessively hungry, forgetful, and caused changes in weight and sexual desire.

*“…… For me I am pregnant, and when I take these medications, I feel they will affect my baby, I have to wait to take them until I deliver.”*(Patient 18, female, 27 years)

**Fear of either taking too many or too few anti-seizure medications**

Patients who had difficulty in using ASMs reported that the medications are overwhelming to be taken per day for the rest of their lives. These patients mentioned that they take more than three types of anti-seizure medications, and they have been using them for more than three years yet missing even one dose makes them get epileptic attacks.

“*I take three types of medications twice daily and have been doing so for four years. However, the moment I miss any dose, I experience an epileptic attack.”…* (Participant 11, male 21 years)

Patients also discussed that there are days when they receive few medications which result into frequent seizures

*“…I have to receive medications for 30 days, this month I have been given for only 15 days, I am worried of getting seizures.”…* (Patient 1, male, 42 years)

## Discussion

This cross-sectional study assessed the prevalence of adherence to anti-seizure medications, factors associated with, and experiences related to ASMs adherence among people living with epilepsy attending a community-based epilepsy clinic in Buikwe District and Mukono General Hospital in Mukono District.

The study revealed that 66.5% of patients adhered to ASMs. In addition, adherence was significantly associated with being aged 30–34 years, earning a monthly income of USD 28 and above, longer waiting time and receiving an inadequate dosage of medications. The qualitative findings further revealed that experiences such as fear of the poor outcomes of epilepsy, social support, financial burden, and individual motivation promoted adherence, whereas financial burden, inadequate knowledge about ASMs, misconception about treatment, community beliefs in traditional healing, fear of medication side effects, and concerns about taking multiple medications contributed to low adherence.

The prevalence of adherence (66.5%) observed in this study is similar to the 62.5% reported in a cross-sectional study among adult patients attending hospital care in Ethiopia [36]. However, it is higher than findings from a multi-country African study involving: Uganda, Kenya, Tanzania, Ghana, South Africa, as well as a study in Ethiopia which reported adherence of 34.9%and 36.5%, respectively [17,37]. The prevalence reported in the current study was lower than 79.5% reported among patients attending a specialized neurology clinic at Mulago National Referral Hospital [38].

These variations may be explained by the differences in sample sizes, study population, study design, study settings, adherence measurement tools, and access to specialized epilepsy care. Unlike tertiary referral facilities, which often manage patients with more severe diseases and provide follow-up services [39], the current study included participants receiving care from both a community-based clinic in a rural setting, and a general hospital. Patients living in rural areas have limited access to health facilities, thus less likely to adhere to ASMs compared to those residing in urban areas that most likely stay close to health facilities, and can afford treatment prescribed, as most of the prescribed medications are procured out of pocket in Uganda [40]. The higher prevalence of adherence to ASMs observed in this study compared to findings from several other studies may have been attributed to contextual and behavioral factors unique to the study population. Participants in this study setting may have high perceptions about the seriousness of epilepsy and benefits of consistent ASMs use, reinforced by strong family and community support, additionally close patient provider interaction and regular follow-up at the facilities may have enhanced patient’s understanding of adherence importance [41]. Additionally, the consistent availability of ASMs at the facilities and the relatively easy access to treatment may have encouraged patients to adhere more to the prescribed regimens [42]. When patients can readily access their medications without frequent stock outs, long distance travel or high costs, they are more likely to utilize their medications well, in contrast in some developed countries, despite advanced health care systems, factors such as complex prescription procedures and high treatment costs may limit consistent access and lead to lower adherence [43]. The higher prevalence may also be attributed to the young age distribution of the study participants [44]. Younger individuals often receive stronger support from their siblings, demonstrate greater adaptability to treatment routines, and demonstrate better cognitive abilities to remember medication schedules [45].

Regarding factors associated with adherence to anti-seizure medications, this study found a lower prevalence of adherence to anti-seizure medications among PLWE aged 30–34 years than those in other age categories. Patients aged 30–34 years are more independent in deciding to take ASMs than those younger than 30 or older than 34 years, who may often rely on relatives for health care support [46]. Additionally, PLWE 30–34 years who are working may have limited time to attend the clinic appointments [47].

Waiting for three or more hours before receiving medications was another factor associated with lower prevalence of adherence to anti-seizure medications. Long waiting times may discourage patients due to frustration with the service, which can stem from factors such as a perceived shortage of healthcare providers, late arrival of staff, high patient volumes, and the presence of severe cases requiring extra attention [48]. Prolonged waiting reduces patients’ willingness to return for epilepsy care, ultimately leading to lower adherence to ASMs. This finding is consistent with findings from a multisite study in Sub-Saharan Africa, western Uganda, and a study conducted in Kenya [49–51].

The study further found a higher prevalence of adherence to ASMs among patients earning at least USD 28 per month. This finding highlights the important role of socioeconomic status in the management of epilepsy [52]. Individuals with higher incomes are better able to afford transport to health facilities, pay for diagnostic tests, and purchase medications when stock-outs occur, as well as maintain better nutrition [53]. This finding is consistent with a study conducted in Singapore [54].

This current study also found that the prevalence of adherence to ASMs was lower among patients who received inadequate dosages. This finding may reflect challenges within the health care system, including medication shortages; weak supply chain management, and inconsistent medication distribution. This may undermine treatment effectiveness, and reduce motivation to adherence [55,56]. This finding is similar with the WHO epilepsy report of 2023, and the global status report on neurology, 2025, which shows that three out of four (3/4) patients with epilepsy in low and middle income countries do not receive the required treatment [57,58].

Regarding experiences on adherence, patients reported that fear of poor consequences of epilepsy motivated them to take their anti-seizure medications consistently. Many had previously experienced seizure recurrence after missing doses and understood the consequences associated with uncontrolled epilepsy. Such experiences appeared to strengthen their commitment to treatment. This finding is similar to a study in Ghana which reported that people living with epilepsy take ASMs to relieve convulsive symptoms associated with the disease [59].

Similarly, fear of stigmatization motivated some patients to adhere to treatment to avoid seizure episodes that could expose their condition and result in discrimination or social exclusion. Given the persistent stigma surrounding epilepsy in many communities, adherence may represent an important strategy for maintaining social acceptance and participation in everyday activities [60]. This finding is consistent with a study conducted in Singapore by Chew and colleagues [61], which reported that people living with epilepsy experience social exclusion, affecting their participation in the society.

Social support was another experience on adherence. Participants described receiving reminders to take medications, financial assistance, emotional encouragement, food support, and support with clinic attendance from family members, neighbors and caregivers. This support helped them to overcome barriers to treatment and maintain consistent medication use. This finding is similar to a study done in Mbale district, Uganda [62], which reported that patients living with epilepsy need consistent support from parents.

On the other hand, poor adherence was associated with inadequate knowledge about ASMs, misconceptions about epilepsy, and community beliefs in traditional healing practices. Some patients doubted the effectiveness of ASMs and preferred traditional remedies because they believed epilepsy was caused by witchcraft or spiritual powers. Such beliefs may discourage patients from using ASMS consistently and contribute to treatment interruptions. Similar findings were reported in studies from Uganda [13,63].

Financial burden was another commonly reported barrier to adherence. Patients described challenges related to transport costs, medication expenses during stock-outs, and loss of income due to epilepsy-related limitations. The economic burden of epilepsy may be particularly severe in low-resource settings, where patients often rely on out-of-pocket payments for healthcare access [64].This finding was also reported by a study in northern Uganda on household poverty among school going children with epilepsy [65], and a study on seizure severity on quality of life among patients living with epilepsy attending outpatient clinic at Mulago National Referral Hospital [66]. Finally, fear of medication side effects and concerns about taking multiple medications contributed to poor adherence. Patients who experienced unpleasant side effects or perceived their treatment regimens as burdensome were more likely to skip doses. The finding was also reported by studies on experiences of medication use in Uganda and South Africa [67,68]. Therefore, the findings of this study demonstrate that adherence to ASMs among PLWE is influenced by a complex interaction of individual, socioeconomic, cultural, and health-system factors. Improving adherence will require multifaceted interventions that address medication availability, patient education, stigma reduction, social support, and economic barriers. Such interventions have the potential to improve seizure control, reduce epilepsy-related complications, and enhance the quality of life of people living with epilepsy in Uganda.

### Integrating the quantitative and qualitative findings

Quantitatively 184 (66.5%) of participants adhered to ASMs, with higher adherence observed among those earning higher monthly incomes, while low adherence was associated with inadequate dosages, longer waiting time and being aged 30–34 years. Qualitative findings complimented these findings by revealing that adherence was influenced by fear of poor consequences of epilepsy, individual motivation, and social support, whereas financial burden, misconception about medications, and lack of awareness about ASMs effectiveness hindered consistent use. These findings suggest that although economic burden and health-system related factors play a significant role in determining adherence levels, patient’s perceptions, social contexts, and motivation strongly shape ASMs adherence in daily life. Increasing knowledge and epilepsy control among PLWE in these settings would help improve drug adherence in the community.

## Strength and limitations of the study

Study strengths: First, in this current study we employed the standardized 8- item Morisky medication adherence scale to minimize patient recall bias that may occur in determining the level of adherence to anti-seizure medications, thus enhancing the quality of the study. Second, we conducted this study both in the urban hospital and community rural settings, which gives it a strong power of Generalizability to rural and urban health care settings in Uganda.

Finally, we used both quantitative and qualitative data collection methods, thus providing a better understanding of adherence to ASMs than either approach alone. By using both methods, a high degree of comprehensiveness of adherence to ASMs can be achieved.

This study however had several limitations. First, the study excluded patients who were severely ill during data collection, yet this could have been caused by low adherence to ASMs, this therefore could have introduced selection bias. Second, the study used a non-probability consecutive sampling procedure which might have led to selection bias. Third, recall bias among participants due to the assessment of their medication history in the past 6 months. Fourth, we conducted this study based on patient perspectives, yet ASMs are provided by health care providers, hence patients have no control of which ASMs to receive. Fifth the MMAS-8 has not been previously validated among epilepsy patients in Uganda. However, this tool has been validated among antihypertensive patients attending outpatient department in Uganda. Sixth, the wide range of ages could introduce bias in the results. Finally, adherence to anti-seizure medications was solely self- reported which might have led to social desirability bias.

## Conclusions

Our findings highlight the need to strengthen collaboration between patients, caregivers, and health care providers by engaging People Living with Epilepsy in education, social and psychological counseling, and offering practical support such as meals and transport to improve medication adherence. Facilities should introduce ASM adherence monitoring tools and routinely document monthly seizure frequency to guide more effective treatment. Community sensitization on the importance of adherence is also essential. To address study limitations, future research should incorporate objective measures such as blood medication levels and use probability sampling to reduce selection bias. Additionally, studies involving both patients and health care providers would offer a shared understanding of adherence, given providers’ critical role in determining treatment options.

## Supporting information

S1 Checklist Checklist. (DOCX)

S2 Data Quantitative data. (XLSX)

S3 Data Qualitative data. (ZIP)
