# Supplementary material for: Adherence to anti-seizure medications among persons living with epilepsy attending a community- based epilepsy clinic in Buikwe and Mukono Districts-Uganda: A mixed methods study
Source: PLoS One. 2026 Aug 21;21(8):e0356494. doi: 10.1371/journal.pone.0356494 (PMC13496452; doi:10.1371/journal.pone.0356494)
Supplement: Supplementary file 2 [file pone.0356494.s005.docx]

**1. Reviewer #1: When reviewing the manuscript, one of the first aspects that requires attention is the consistency in formatting and reference style, particularly the placement of citations. It is important to clearly decide whether there should be a space before the references and to ensure that this is applied uniformly throughout the entire text. Consistent referencing improves readability and professional presentation, which is particularly relevant in a journal like PLOS ONE.**

Formatting and referencing style has been corrected

**2. With respect to the methodological aspects, I would appreciate further clarification regarding the recruitment process of the study participants. Specifically, how exactly were patients approached and invited to participate in the study? Were they directly asked by a clinician or research assistant if they wished to join the study, or was another strategy employed? It would be valuable to know whether data were collected on patients who declined participation. If such information exists, it could provide an important perspective on potential recruitment bias. Moreover, describing the clinical and socio-economic features of those who did not agree to participate would offer useful insights into the Generalizability of the study findings.**

This has been addressed as follows;

- **How exactly where patients approached and invited to participate in the study?**

Participants were approached through the mental health care workers and the responsible community health workers who introduced the research team to the study respondents.

- **It would be valuable to know whether data were collected on patients who declined participation**

During data collection, 2 respondents who were; a male and a female aged 30 and 50 years respectively from urban areas, and attending to Mukono General Hospital mental health clinic declined participation in the study.. Data was not collected from these respondents.

**3. Another important element relates to the language and validation of the questionnaire. In which language was the Morisky Medication Adherence Scale administered? Was this version formally validated for use in this population, and if so, could the authors provide the reference to the validation study or describe the adaptation process? If not, it may be important to acknowledge this as a potential limitation**

**Language the tool was administered**

The tool was administered in Luganda, the language used in Buikwe and Mukono districts. This has been stated as follows: Two translators independently translated the English version of the MMAS-8 to Luganda, the dominant widely used language in Buikwe and Mukono districts, Uganda. A third translator performed reverse translation from Luganda to English. The original and back translated versions were compared and inconsistencies were resolved. A pilot test was performed in 10 patients with epilepsy and the interpretation of the tool was consistent. The patients who participated in this face- validity did not participate in this study.

- **Validation of the MMAS-8 in this population**

The MMAS-8 has been applied in studies measuring ASMs in Uganda but its specific validity for this use is derived from its general validation for chronic diseases. For hypertensive patients, the factor analysis of construct validity of the scale has been previously proven to be good with overall Kaiser’s measure of sampling adequacy for residuals of 0.72, and moderate internal consistency (Cronbach α=0.65). The MMAS-8 is one of the most widely used tools to measure medication adherence and has been validated in a wide range of population groups. References have been added to support this statement.

The MMAS-8 has not been previously validated among epilepsy patients in Uganda. However this tool has been validated among antihypertensive patients attending outpatient department in Uganda

This has also been reported as a limitation for this study

**4. Regarding the section on “Characteristics of the study respondents”, I would recommend providing comparative data from the general population or from available regional statistics. Such a comparison would allow the reader to better understand how representative or distinctive this cohort is in relation to the broader population. It would also strengthen the epidemiological interpretation of the findings.**

A statement on comparative data has been added in the introduction section as follows;

The national wide crude prevalence of epilepsy in Uganda is 16.9 per 1000 persons with an age- and sex- prevalence rates of 17.8 per 1000 among all individuals from one month to 108 years

**5. In the section titled “Characteristics of respondents for the qualitative study”, there appear to be several typographical and spacing errors, particularly around punctuation marks. A careful language and formatting review of this section is advised to ensure clarity and precision**.

Punctuation and language review has been done for this section

**6. The finding of a surprisingly high adherence rate in this cohort compared to other studies conducted in similar resource settings, as well as in some high-income countries, deserves deeper reflection and contextualization. One possible explanation may be the younger age distribution of the sample, which could be associated with stronger family involvement, including support from parents or siblings. It may also be relevant to consider the role of community support systems, health worker–patient relationships, or programmatic factors such as medication availability and accessibility.**

**In addition to these points, I would suggest the authors consider a few further clarifications and expansions**

This has been expanded as follows;

The higher prevalence of adherence to ASMs observed in this study compared to findings from several other studies may have been attributed to contextual and behavioral factors unique to the study population. Participants in this study setting may have high perceptions about the seriousness of epilepsy and benefits of consistent ASMs use, reinforced by strong family and community support, additionally close patient provider interaction and regular follow-up at the facilities may have enhanced patient’s understanding of adherence importance.

The consistent availability of ASMs at the facilities and the relatively easy access to treatment may have encouraged patients to adhere more to the prescribed regimens. When patients can readily access their medications without frequent stock outs, long distance travel or high costs, they are more likely to utilize their medications well, in contrast in some developed countries, despite advanced health care systems, factors such as complex prescription procedures and high treatment costs may limit consistent access and lead to lower adherence.

The higher prevalence of adherence to ASMs observed in this study may also be attributed to the young age distribution of the study participants. Younger individuals often receive stronger support from their siblings, demonstrate greater adaptability to treatment routines, and demonstrate better cognitive abilities to remember medication schedules

**7. It would be beneficial to provide more information on whether adherence was cross-checked through any objective measure (e.g., pill counts, pharmacy refill records, or serum levels) or whether it relied solely on self-report. Even if not available, a brief reflection on this limitation would be appropriate.**

This has been addressed as a limitation of the study and stated as follows;

Adherence to anti-seizure medications was solely self- reported which might have led to social desirability bias. We recommended a further study focusing on using blood levels other than self-report in determining adherence to ASMs would produce more quality results

**8. The authors may wish to describe how missing data were handled during analysis and whether there was any imputation or sensitivity testing**

This has been added in the manuscript and stated as follows;

Data were sorted, edited and entered into excel sheet. Data cleaning was then done to ensure consistence and completeness.

Additionally, research assistants were adequately and regularly supervised. The supervision entailed checking on the completeness and accuracy of the completed data collection forms.

**9. Given that long waiting times and treatment adequacy were identified as factors associated with adherence, it could be helpful to expand the discussion on structural or health system factors that might influence adherence in this setting.**

These have been addresses as follows;

Longer waiting time often reflects systematic inefficiencies such as staff shortages, inadequate patient flow management and overcrowded clinics. These challenges can discourage patients from attending to the clinics or refilling prescriptions, ultimately lowering adherence to ASMs among people with epilepsy.

Structural and health system related factors can influence the quality of services provided to patients. There is limited drug supply, poor procurement procedures, and inconsistent distribution of drugs which contribute to patients receiving inadequate dosages which can undermine treatment effectiveness and reduce motivation to adherence

**10. Since the study uses a mixed-methods approach, a clearer description of how the qualitative and quantitative findings were integrated could strengthen the methodological rigor of the paper**.

A section on integrating the research findings has been added and described as follows;

Quantitatively 66.5% of participants adhered to ASMs, with higher adherence observed among those earning higher monthly incomes, while low adherence was associated with inadequate dosages, longer waiting time and being aged 30 to 34 years. Qualitative findings complimented these findings by revealing that adherence was influenced by fear of poor consequences of epilepsy, individual motivation, and social support, whereas financial burdens, misconception about medications, and lack of awareness about ASMs effectiveness hindered consistent use. These findings suggest that although economic burden and health system related factors play a significant role in determining adherence levels, patient’s perceptions, social contexts, and motivation strongly shape ASMs adherence in daily life.

**Additional Editor Comments:**

There are some remarks about this manuscript
**1: There are typing errors and abbreviations without explanation**

We have corrected the typing errors and revised the abbreviations

**2: More details need about the study design**

More details have been added on the study design as follows;

**Study design and population**

The study was a cross-sectional design conducted both in the community and at the hospital, employing quantitative and qualitative methods. The study adhered to the Strengthening the Reporting of Observational Studies in Epidemiology (STROBE) guidelines. The study population consisted of Persons living with epilepsy 15 years and above attending regularly at the community-based epilepsy clinic in Buikwe District, and Mukono General Hospital, Mukono District. The study was carried from 05^th^ April to 20^th^ July 2024. Participants were approached through the mental health care workers, and the community health workers who introduced the research team to the study respondents.

**3: A wide range of age will bias the results, particularly with studies on the epilepsy and medication adherence**

We have explained this as follows;

First, we have re-categorized age from a range of 10 to 5 years to eliminate bias of the results. Secondly, Epilepsy being a rare condition in our settings, we recruited participants from 15 years and above in order to meet the sample size. Thirdly, the number of patients aged 15 to 19 years is reportedly high in these settings. Recruiting patients aged 15 and above has also been considered as a limitation of the study
**4. The sample size was not calculated**

We have indicated the details of how the sample size was calculated as follows:

For quantitative data, a sample size of 277 was determined using the modified formula for cross-sectional studies by Kish- Leslie 1965

N = z^2^p (1-p)/d^2^

Where N is the sample size

d = level of precision/ error, a precision of 5%

z = standard normal deviation corresponding to a 95% confidence interval, 1.96

p = the assumed probability of patients with adherence to anti-seizure medications; p=0.209

N= (1.96^2^ x 0.209x0.791)

0.05^2^

= 254

To account for potential non response, an additional 10% were included

(254*0.1) + 254

=	279

During data collection, 2 respondents who were; a male and a female aged 30 and 50 years respectively from urban areas, and attending to Mukono General Hospital mental health clinic declined participation in the study. Therefore; 277 study participants were enrolled for the quantitative part of the study

**5: The validity of the questionnaire is not clear and not reported**

The validity of the questionnaire has been explained and elaborated as follows;

The MMAS-8 has been applied in studies measuring ASMs in Uganda but its specific validity for this use is derived from its general validation for chronic diseases. For hypertensive patients, the factor analysis of construct validity of the scale has been previously proven to be good with overall Kaiser’s measure of sampling adequacy for residuals of 0.72, and moderate internal consistency (Cronbach α=0.65). The MMAS-8 is one of the most widely used tools to measure medication adherence and has been validated in a wide range of population groups. References have been added to support this statement.

The MMAS-8 has not been previously validated among epilepsy patients in Uganda. However this tool has been validated among antihypertensive patients attending outpatient department in Uganda

This has also been reported as a limitation for this study

**6: The Cronbach's alpha is not calculated**

Cronbach’s alpha has been calculated as indicated in the equation below

**Calculating the Chronbach’s Alpha**

α=(K)/(K-1)*(Sy^2^-∑Si^2^)/Sy^2^)

Where;

α= Chrombach’s alpha

K=number of items in the scale

Si=sum of item scores for each item

S=sum of total scores for all the items

α= (8/8-1)*(2216^2^-1477^2^)/2215^2^

α=0.64

**7: The level of significance 0.2?????? whether one or two-tailed?s**

This has been corrected and stated as follows

A p-value threshold of 0.05 was used to assess for statistically significant associations

**8: Tables are not well prepared; the headings and subheadings of the variables were in separated rows**.

The headings and subheadings of the variables have been put in the same rows

**9: The data of Morisky-8 and its analysis are not reported**

Data of the MMAS-8 and how it was analyzed has been included

**10: The results of the Table 3 are not informative**

To make the contents of table 3 more formative, we have included text information instead of the table

**11: Statistical analyses are inappropriate**

Statistical analysis has been described further for clarity

**12: References are not like the Style of the Journal**

References have been changed to the style of the journal
