# Supplementary material for: Adherence to anti-seizure medications among persons living with epilepsy attending a community- based epilepsy clinic in Buikwe and Mukono Districts-Uganda: A mixed methods study
Source: PLoS One. 2026 Aug 21;21(8):e0356494. doi: 10.1371/journal.pone.0356494 (PMC13496452; doi:10.1371/journal.pone.0356494)
Supplement: Supplementary file 3 [file pone.0356494.s003.zip › IDI 1.docx]

**Participant one**

**Interviewer**: The next step is to know your understanding about adhering to anti-seizure medications. So, what do you know about adhering to anti-seizure medications?

**Respondent**: It means taking medicines as given by doctors

**Interviewer**: OK, so with regards to knowing how to adhere to anti-seizure medications, how did you get to know about adhering to anti-seizure medications?

**Respondent**: I was brought in by my father to the clinic; I was encouraged to take the drugs from there by the doctors

**Interviewer**: All right. So, how did you start adhering to anti-seizure medications?

**Respondent**: Oh, because of seeing others taking, I also started taking medication as their lives had improved so much, and they were looking normal

**Interviewer**: Tell me those things that prompt you to adhere to anti-seizure medications?

**Respondent**: Oh, first not to be seen by people that I fall daily when at home, second I also want to be proud, and to participate in basic activities at home like any other person, also as for me, when I fail to take my drugs, I will be most likely to fall while in public and people will get to know that I have epilepsy, I therefore have to take them on time

**Interviewer**: So, our next question concerns the good things about adhering to anti-seizure medications. Are there good things that you have to say about anti-seizure medications for the time you have been adhering to it?

**Respondent**: Oh, yes, they are good for me, let me continue taking the drugs, I am so interested in taking them but they are also many

**Interviewer**: Ok, how do other people with epilepsy experience adhering to anti-seizure medications? Have other people seen benefits of adhering to these medications.

**Respondent**: Yes, others take them, but I am not so sure how, but may be they like them, sure this is seen at the clinic on how they keep advising us to take the medications

**Interviewer**: I would like to know the advantages of adhering to anti-seizure medications, what are they?

**Respondent**: They stop you from falling when taken on time, they are better than the herbals as are not so big, but are tasteless

**Interviewer**: Ok, then what are the challenges that people who adhere to anti-seizure medications face

**Respondent**: Most people do not take them daily, they lack money, some medications are also very expensive to buy, if you are not working, again not taking medications is dangerous. ….also even if we use these medications as instructed, sometimes health workers bring those that have lasted for long, they tend to look more Powderly and different from the ones always provided, whenever you swallow them, no improvement is seen but get more attacks. Like today, I have to be receive medications for 30 days.., but they have given me for only 15 days, I am likely to get seizures

**Interviewer**: I am sure we need to advocate, our next question is, tell me the community views about adhering to anti-seizure medications

**Respondent**: They say we are not good people, so the medications cannot work; we need to behave well in communities. Some say we need consistent support, they also say that I always take my medications as instructed by the health care providers, but I get seizures and fall in a way similar to how a thief that was stealing yellow bananas from my grand fathers garden fell when he was shoot with a spear by the grandfather, such falls have not reduced even with taking these drugs for many years

**Interviewer**: How can adhering to anti-seizure medications anti-seizure medications be improved?

**Respondent**: Giving more medications is the best plan, sometimes we do not get medications because they are not there. Like me I didn’t get…enough, last time, may be they are not there in their stock, medications given are also not enough I think

**Interviewer**: Ok, considering the previous days, tell me the days that you have adhered to your anti-seizure medications in the last five days

**Respondent:** All days if I have but last week didn’t have them, but I ensure to take all my medications well as being given

**Interviewer**: I would like to know the kinds of anti-seizure medications that you know, what are they

**Respondent**: Oh, I don’t know their names, but there is a yellow, two round medications, I am not familiar with their names but I take those ones always

**Interviewer**: How would you recommend a person with epilepsy to adhere to medications anti-seizure medications?

**Respondent**: To take like doctors say, we people should not play with these medications, they can work well

**Interviewer**: Looking at the current available guidelines in adhering to medications which include; eating on time, taking the required dosage and avoiding missing the dosage. How would you comment on the current available guidelines concerning adherence to anti-seizure medications?

**Respondent**: They are good but medications are not enough

Thank you for participating in this interview
