# Supplementary material for: Adherence to anti-seizure medications among persons living with epilepsy attending a community- based epilepsy clinic in Buikwe and Mukono Districts-Uganda: A mixed methods study
Source: PLoS One. 2026 Aug 21;21(8):e0356494. doi: 10.1371/journal.pone.0356494 (PMC13496452; doi:10.1371/journal.pone.0356494)
Supplement: Supplementary file 3 [file pone.0356494.s003.zip › IDI 2.docx]

**Participant 2**

**Interviewer**: What do you know about adhering to anti-seizure medications?

**Respondent**: Oh, yes it means taking all the medications as being given by a qualified doctor so as to be healthy

**Interviewer**: Ok, how did you get to know about adhering to anti-seizure medications?

**Respondent**: So, my sister, she brought me to take, and be on, so she has always been telling me to keep using them well

**Interviewer**: So looking at any available factor, how did you start adhering to anti-seizure medications?

**Respondent**: For me I just take them, as like I am saying, sometimes I just like to take them as they have no problem, so I keep taking as many times as I can so long as I follow instructions

**Interviewer**: Al right, tell me those things that prompt you to adhere to anti-seizure medications?

**Respondent**: People want me to take them, not to disturb them, sometimes I could disturb people and could take me to the hospital or even pick me from the roadside when I fall, may be that is why they want me to take the medications

**Interviewer**: Looking at the good things about anti-seizure medications. Are there things that you have to say about anti-seizure medications for the time you have been adhering to it

**Respondent**: These medications, give me headache feel like living them, may be just have to continue with them, I would give up but may be will again become sick, let me keep taking all of them

**Interviewer**: Oh, thank you, how do other people with epilepsy experience adhering to anti-seizure medications?

**Respondent**: I am not sure, but I think they are feeling good, sometimes I see them looking at us when going for medications and give us some banana or eats

**Interviewer**: I would like to know the advantages of adhering to anti-seizure medications, what are they?

**Respondent**: To be silent and to be like others, they don’t want us to confuse them, especially at home and at work

**Interviewer**: What are the challenges that people who adhere to anti-seizure medications face

**Respondent**: They make me eat a lot of food; those always make me thirsty, also sometimes not taking them makes you become weak. Also when I am pregnant, I fear to take these drugs, I know that they can may be affect my baby, I have to wait taking them until I deliver

**Interviewer**: Tell me the community views about adhering to anti-seizure medications

**Respondent**: They say no problem, I think they are ok, they looked at me when got it in class. When I had not taken my medication, I got an attack while in class, everyone including the teacher ran out of class, when I was awake, nobody was in class and everyone was seeing me from the windows, I stopped going to school

**Interviewer**: Oh that was challenging, how can adhering to anti-seizure medications be improved?

**Respondent**: Having enough rest and meals I think, they need eat a lot, without eating makes you feel dizzy always

**Interviewer**: Tell me the days that you have adhered to your anti-seizure medications in the last five days

**Respondent**: Every day I take my medicines, they support me, so I like my parents and the community members who encourages me

**Interviewer**: I would like to know the kinds of anti-seizure medications that you know, what are they

**Respondent**: They are; big, white with no other color, but are better medications for me. The names I am not sure

**Interviewer**: Considering other people who use these medications, how would you recommend a person with epilepsy to adhere to medications anti-seizure medications?

**Respondent**: To eat well daily after taking the medicines as they are heavy, and cause appetite, so one has to always eat a lot of food to be healthy

**Interviewer**: There are guidelines by the health care providers regarding adhering to drugs. How would you comment on the current available guidelines concerning adherence to anti-seizure medications?

**Respondent:** They are there but few, medicines are expensive, some are difficult to swallow, have big sizes and requires support

**Interviewer**: Thank you very much for your participation
