# Supplementary material for: Adherence to anti-seizure medications among persons living with epilepsy attending a community- based epilepsy clinic in Buikwe and Mukono Districts-Uganda: A mixed methods study
Source: PLoS One. 2026 Aug 21;21(8):e0356494. doi: 10.1371/journal.pone.0356494 (PMC13496452; doi:10.1371/journal.pone.0356494)
Supplement: Supplementary file 3 [file pone.0356494.s003.zip › IDI 3.docx]

**Participant 3**

**Interviewer**: What do you know about adhering to anti-seizure medications? What does it mean?

**Respondent**: I think to take my, medications such that I don’t fall, and for people, not to see me even if I am with them, I think that is what it means, also taking all the medications given to patients

**Interviewer**: Ok, our next question is; how did you get to know about adhering to anti-seizure medications.

**Respondent**: I was told by my father, that I have to take my medications; he also insisted that I take them well as it is given to me, but the mum also wants me to take them

**Interviewer**: So, your parents helped you. Then how did you start adhering to anti-seizure medications?

**Respondent**: didn’t like but just take, the parents are strict and always wanted, to see that I take all the medications, maybe this was also good

**Interviewer**: Tell me those things that prompt you to adhere to anti-seizure medications?

**Respondent**: Yes, the important thing is to be alive and to work since, I want to support myself for food so let me always take the medications daily

**Interviewer**: Are there things that you have to say about anti-seizure medications for the time you have been adhering to it? Do these medications work?

**Respondent**: They don’t work, I just take but they do not work, I think if they were working, for the time I have taken them, I would have healed by now

**Interviewer**: Considering other people living with epilepsy, how do they experience adhering to anti-seizure medications? How are they

**Respondent**: Medications don’t work for them, they are so heavy, I keep hearing them saying that whenever I go to the clinic, yes they are heavy because you cannot do any work after taking them, especially those receiving three types. So you have to eat and drink in order to work

**Interviewer**: I would like to know the advantages of adhering to anti-seizure medications, what are they?

**Respondent**: Oh, thank you, I think to stop falls but they still come, to be with others and help each other at home, falls can reduce when medications are taken especially at the best time, in the day

**Interviewer**: Are there challenges that people taking these medications face? What are the challenges that people who adhere to anti-seizure medications face

**Respondent**: Giving many medications to swallow, big sizes are a problem, but we have to take them, some even lead to excess salivation. Also because of the costs, I miss picking my medications sometimes, like last time, so it becomes difficult to use them well

**Interviewer**: Considering the community where you stay, tell me the community views about adhering to anti-seizure medications

**Respondent:** They think medications don’t work all the time, they think we have problems, that is what they say but I don’t know if they are correct, but also if mum is not there, I cannot miss getting my medications as I’m always helped to pick them for free by a motorcycle rider, I always inform him a day before the clinic day, such that when he is going to do his work in town, we go together and I pick my medications such that I can take them as instructed

**Interviewer**: How can adhering to anti-seizure medications be improved?

**Respondent:** Bringing more doctors, especially in morning hours, the doctors we have are few to help us all, you see the numbers are also increasing hence need more doctors to see us

**Interviewer**: Tell me the days that you have adhered to your anti-seizure medications in the last five days

**Respondent**: For me I have taken my medications all the days this week, but last week I missed, but I have consistently been taking them

**Interviewer**: I would like to know the kinds of medications anti-seizure medications that you know, what are they

**Respondent**: Many medications, four of them, they are yellow, white, but also big in color

**Interviewer**: How would you recommend a person with epilepsy to adhere to medications anti-seizure medications?

**Respondent:** To also seek more care from other clinics, may be also to continue taking the them well, we shall be fine

**Interviewer**: How would you comment on the current available guidelines concerning adherence to anti-seizure medications?

**Respondent:** I think they need to be improved; they are not helping very well

**Interviewer**: Thank you for participating
