# Supplementary material for: Adherence to anti-seizure medications among persons living with epilepsy attending a community- based epilepsy clinic in Buikwe and Mukono Districts-Uganda: A mixed methods study
Source: PLoS One. 2026 Aug 21;21(8):e0356494. doi: 10.1371/journal.pone.0356494 (PMC13496452; doi:10.1371/journal.pone.0356494)
Supplement: Supplementary file 3 [file pone.0356494.s003.zip › IDI 4.docx]

**Participant 4**

**Interviewer**: Our first question is, what do you know about adhering to anti-seizure medications?

**Respondent**: Taking the medications as they are give them to me, it also means taking the anti-seizure medications as they are available

**Interviewer**: I am also interested in how you got to know about adhering to anti-seizure medications. How did you get to know about adhering to anti-seizure medications?

**Respondent**: So, the parents said they called me, was not behaving well, they say I was difficult to control, those called me up to take the medications

**Interviewer**: How did you start adhering to anti-seizure medications?

**Respondent**: My sisters wanted me to take them, so they encouraged me to take the mediations daily, l do not feel happy if I fail to take the medications, but these days even the doctors encourage me to take them

**Interviewer**: Tell me those things that prompt you to adhere to anti-seizure medications?

**Respondent**: You know, the medications are there, I need to have good life, I don’t want people to know that thing hits me and fall, they may run away

**Interviewer**: Are there things that you have to say about anti-seizure medications for the time you have been adhering to it

**Respondent**: Yes, after swallowing them, I feel weak sometimes, and sleep off, if I do not have food, sometimes I feel the head is heavy

**Interviewer**: How do other people with epilepsy experience adhering to anti-seizure medications?

**Respondent**: I don’t know, but some think we are wasting time on medications, medications do not work well…..they say that, we just take

**Interviewer**: I would like to know the advantages of adhering to anti-seizure medications, what are they?

**Respondent**: True, People start liking you, you are supported by the family and you …. are happy every day, you do not become sick every time

**Interviewer**: What are the challenges that people who adhere to anti-seizure medications face

**Respondent**: When you don’t take, you may fall off, like as if you are not normal, I was climbing a tree….fall got injury, so don’t like missing

**Interviewer**: Tell me the community views about adhering to anti-seizure medications

**Respondent**: They give me some food, sometimes give free ride, to the clinic, so as I bring the medications, they like to support me if they have something

**Interviewer**: How can adhering to anti-seizure medications anti-seizure medications be improved?

**Respondent**: They need to teach the people, all should be taught about the benefits of the medications as…..they work, that is good, also create more awareness

**Interviewer**: Tell me the days that you have adhered to your anti-seizure medications in the last five days

**Respondent**: I missed one day, my sisters ensures that I take them daily, they like to give me food, I tell you we have to always eat on time, that may work

**Interviewer**: Specifically looking at the medications, I would like to know the kinds of anti-seizure medications that you know, what they are

**Respondent**: I don’t know, they are many but sore, itchy when taking but just have to take, ok what I remember is I take 3 types, a round white big medication and a square one

**Interviewer**: How would you recommend a person with epilepsy to adhere to medications anti-seizure medications?

**Respondent**: To take them, even in if they are sour, we need life, every patient with epilepsy should take them, just continue taking them I think.

**Interviewer**: How would you comment on the current available guidelines concerning adherence to anti-seizure medications?

**Respondent**: The medications which are brought expensive, they need to sensitize us, they need when you have money as are expensive if missed, it is also difficult to find where to buy them

**Interviewer**: That is the end Thank you for participating in this interview
