# Supplementary material for: Adherence to anti-seizure medications among persons living with epilepsy attending a community- based epilepsy clinic in Buikwe and Mukono Districts-Uganda: A mixed methods study
Source: PLoS One. 2026 Aug 21;21(8):e0356494. doi: 10.1371/journal.pone.0356494 (PMC13496452; doi:10.1371/journal.pone.0356494)
Supplement: Supplementary file 3 [file pone.0356494.s003.zip › IDI 5.docx]

**Participant 5**

**Interviewer**: Please tell me, what you know about adhering to anti-seizure medications?

Respondent: It means taking medications every day, especially after eating food

**Interviewer**: Considering knowledge about adhering to anti-seizure medications, how did you get to know about adhering to anti-seizure medications?

**Respondent**: For me I didn’t know, so I could just take them, I have been taking them since childhood, I started taking them when I was not aware, so I am not so sure how I got to know

**Interviewer**: How did you start adhering to anti-seizure medications?

**Respondent:** I don’t know about that but I used to see other people coming for the medications at the clinic, and my parents also supported me to start taking them by accompanying me to the clinic

**Interviewer**: Tell me those things that prompt you to adhere to anti-seizure medications?

**Respondent**: I want to continue working, and not to fall in fire while cooking food, so I have to take the medications well, also to have good life like others. You know I need my health and do not want to get any physical disability that may worsen my health; the only way I can have my health is by taking medications as instructed by the health care providers

**Interviewer**: Are there things that you have to say about anti-seizure medications for the time you have been adhering to it. Do anti-seizure medications work?

**Respondent:** I think these medications work, being, I could only use herbals thinking they work better, but I have seen that these medications work better than herbals

**Interviewer**: Ok, then how do other people with epilepsy experience adhering to anti-seizure medications?

**Respondent**: They use other drugs, the herbals which are better, may be if they stop using herbals but for me I think they also like them, you know each of us see these medications differently

**Interviewer**: I would like to know the advantages of adhering to anti-seizure medications, what are they?

**Respondent**: Sometimes reduce falls but they are not there sometimes, yes they reduce falls many times mostly if they are also used with herbals

**Interviewer**: Our next question is on challenges associated with anti-seizure medications. What are the challenges that people who adhere to anti-seizure medications face

**Respondent**: Coming for the on foot, others get, those attacks, when on the way, this may make some of us not consistent at the clinic hence not to using the medications well

**Interviewer**: Tell me the community views about adhering to anti-seizure medications

**Respondent**: We are not being helped, but other motorcycles carry us for free, so they think we have to help each other, others do not bother and just look at us

**Interviewer**: How can adhering to anti-seizure medications be improved?

**Respondent:** Teaching, eating more food, encouraging counseling of the community, as they need to also continue helping us, it is really challenging, so they also have to know

**Interviewer**: Tell me the days that you have adhered to your anti-seizure medications in the last five days

**Respondent:** This week I have not missed any day but I missed last week, as the medications were not enough, as the month was ending, yes sometimes the medications do not finish the whole month thus becoming a challenge to all of the patients

**Interviewer**: I would like to know the kinds of anti-seizure medications that you know, what are they

**Respondent:** Artane, yellow and big tablets, I don’t know their names

**Interviewer**: Oh, then, how would you recommend a person with epilepsy to adhere to medications anti-seizure medications?

**Respondent**: They should always work so as to be able to buy the medications if they miss, you know we always struggle to look for money, if you don’t work you lack what to eat and even the medications

**Interviewer**: How would you comment on the current available guidelines concerning adherence to anti-seizure medications?

**Respondent**: For me I think the best is to be close to your relatives, even at work the family is important. I don’t know about other guidelines

**Interviewer**: Thanks for participating
