# Supplementary material for: Adherence to anti-seizure medications among persons living with epilepsy attending a community- based epilepsy clinic in Buikwe and Mukono Districts-Uganda: A mixed methods study
Source: PLoS One. 2026 Aug 21;21(8):e0356494. doi: 10.1371/journal.pone.0356494 (PMC13496452; doi:10.1371/journal.pone.0356494)
Supplement: Supplementary file 3 [file pone.0356494.s003.zip › IDI 6.docx]

**Participant 1**

**Interviewer**: please, what do you know about adhering to anti-seizure medications?

**Respondent**: It means taking my medications for epilepsy and not to clear life or get recurrent sicknesses, they are our medications, so I think, we need to enjoy them please

**Interviewer**: OK, then how did you get to know about adhering to anti-seizure medications?

**Respondent**: The doctors told us to take them, we have to do so, and use them very well to prevent falls that affect our lives

**Interviewer**: Ok please, then how did you start adhering to anti-seizure medications?

**Respondent**: It started when my parents took me to the clinic, and they told me the medications work so I have to use them, they said the medications are good, so I had to start taking them everyday to be healthy

**Interviewer**: Tell me those things that prompt you to adhere to anti-seizure medications?

**Respondent**: Oh, true, let me tell you, for me I need to ride my motorcycle to earn a living, I need money to support my family, and I also become happy if I work and get some money to do something for myself please

**Interviewer**: What do you say about anti-seizure medications? Are there things that you have to say about anti-seizure medications for the time you have been adhering to it

**Respondent**: Oh yes, after swallowing these medications, you need to eat, otherwise you experience headache, and become weak. Again if I don’t eat I feel dizzy while riding a motorcycle, so I can easily fall, and also the medications are expensive is you miss from the clinic

**Interviewer**: How do other people with epilepsy experience adhering to anti-seizure medications?

**Respondent**: I think they do not take them well, they rely on herbals sometimes, and they get seizures, also most of them complain of many medications given to them

**Interviewer**: I would like to know the advantages of adhering to anti-seizure medications, what are they?

**Respondent**: Yes they prevent you from getting seizures most especially when you are walking. Also people can’t get to know that you experience seizures, and you become at least stable

**Interviewer**: What are the challenges that people who adhere to anti-seizure medications face

**Respondent**: Oh, I think these medications only work for some time, if they…….were working, I would have healed by now…, they are difficult to take, if you are weak, and one can easily fall in hard places.

**Interviewer**: Tell me the community views about adhering to anti-seizure medications

**Respondent**: They say those doctors lie us, they think those medications cause harm to us, it’s better not take them, that we should just change our behavior in the communities, then we shall be ok

**Interviewer**: Thanks for that, we need to work hard and advocate, how can adherence to anti-seizure medications be improved?

**Respondent**: we should be given all the medications, also more sites to distribute the medications should be increased; ok then we can be well

**Interviewer**: Tell me the days that you have adhered to your anti-seizure medications in the last five days, did you take them yesterday

**Respondent**: Yes, yesterday I took them, I always take them most of the days, even yesterday I took, cannot miss. Ok, only that they work slowly

**Interviewer**: Ok, I would like to know the types of anti-seizure medications that you know, what are they

**Respondent**: Oh, I don’t know their names, can I bring them, but a yellow, white round big one and small white, that is what I know, and remember

**Interviewer**: Thank you, how would you recommend a person with epilepsy to adhere to medications anti-seizure medications?

**Respondent**: Let me tell you, one has to be serious with them, need to take after food daily, people should not stop us from taking these medications

**Interviewer**: How would you comment on the current available guidelines concerning adherence to anti-seizure medications?

**Respondent**: I think are good

**Interviewer**: Anything else you want to say about anti-seizure medications

**Respondent**: No

**Interviewer**: Thank you
