# Supplementary material for: Adherence to anti-seizure medications among persons living with epilepsy attending a community- based epilepsy clinic in Buikwe and Mukono Districts-Uganda: A mixed methods study
Source: PLoS One. 2026 Aug 21;21(8):e0356494. doi: 10.1371/journal.pone.0356494 (PMC13496452; doi:10.1371/journal.pone.0356494)
Supplement: Supplementary file 3 [file pone.0356494.s003.zip › IDI 7.docx]

**Participant 7**

**Interviewer**: I would like to know what you understand by adhering to anti-seizure medications.

**Respondent:** Taking my medications for treating the disease, and not to have seizures, and maybe also not to die, I think also in order to be normal, enjoy life I think

**Interviewer**: Then how did you get to know about adhering to anti-seizure medications?

**Respondent:** I was sent by a friend there, oh; I liked him as he was concerned, so for me, he thought I was not behaving well in the community

**Interviewer**: Ok please, then how did you start adhering to anti-seizure medications?

**Respondent:** Yes, I could go to the clinic with my friend to see the doctors, the medications were not enough at first but, I liked them

**Interviewer**: Tell me those things that prompt you to adhere to anti-seizure medications?

**Respondent:** Yes, I needed to be with others, and not to be feared, I also liked to do my income generating activities which is selling fuel to support myself, I need the medications

**Interviewer**: Are there things that you have to say about anti-seizure medications for the time you have been adhering to it. Do the medications work?

**Respondent:** Oh, you need to eat a lot of food as this helps to avoid headache and being sleepy, eating prevents the weakness

**Interviewer**: What about other people with epilepsy, how do they experience adherence to anti-seizure medications?

**Respondent**: I am not sure of what they experience, may be they do not take well, others rely on herbals and other local drugs, so these then get falls daily

**Interviewer**: I would like to know the advantages of adhering to anti-seizure medications, what are they?

**Respondent:** Yes, thank you, they reduce the number of seizures, people do not get to know that you have epilepsy, my family is supportive to pick medications if I am unable.

**Interviewer**: What are the challenges that people who adhere to anti-seizure medications face

**Respondent:** Let me tell you, those medications work, but doctors come late, they have many patients to attend to before they reach here, at time we wait for long hours at the clinic before they come, imagine waiting for 5 hours, but they are concerned doctors

**Interviewer**: Ok, we have to advocate, tell me the community views about adhering to anti-seizure medications

**Respondent:** They say people living with epilepsy are cursed; the medications do not work well, that we also need more support. Sometimes you feel isolated at meetings

**Interviewer**: To you, how can adhering to anti-seizure medications be improved?

**Respondent:** We need to take all the medications if we have them, these medications are expensive, may be even other patients with epilepsy should try using them, they may be good for them

**Interviewer**: Tell me the days that you have adhered to your anti-seizure medications in the last five days, did you take them yesterday

**Respondent:** Yesterday I took the medications, and I took all of them, even when I travel, I have to move with them, I only miss if they are finished like last month I missed for 5 days

**Interviewer**: Please tell me the kinds of anti-seizure medications that you know, what are they?

**Respondent:** Tegretol, and folic acid, they work for me, Tegretal looks good but you need to take it with food

**Interviewer**: Thank you, how would you recommend a person with epilepsy to adhere to medications anti-seizure medications?

**Respondent:** They should stop listening to community rumors, I think the medications work, they are so good, if they need good health, they should use them, people should always take the medications without any fear

**Interviewer**: How would you comment on the current available guidelines concerning adherence to anti-seizure medications?

**Respondent:** They work, all doctors work, and they bring the medications closer

**Interviewer**: Anything else you want to say about anti-seizure medications

**Respondent:** No, thanks for asking

**Interviewer**: Thank you
