# Supplementary material for: Adherence to anti-seizure medications among persons living with epilepsy attending a community- based epilepsy clinic in Buikwe and Mukono Districts-Uganda: A mixed methods study
Source: PLoS One. 2026 Aug 21;21(8):e0356494. doi: 10.1371/journal.pone.0356494 (PMC13496452; doi:10.1371/journal.pone.0356494)
Supplement: Supplementary file 3 [file pone.0356494.s003.zip › IDI 8.docx]

**Participant 8**

**Interviewer**: I would like to know what you understand by adhering to anti-seizure medications.

**Respondent**: It means taking all my medications, as given to me by the doctors, it also means using them daily every month

**Interviewer**: How did you get to know about adhering to anti-seizure medications?

**Respondent**: My mother took me to the clinic and she likes me when I am taking the drugs. I have to keep taking them, so I got to know about those medications through my mother, and they are working

**Interviewer**: Please, how did you start adhering to anti-seizure medications?

**Respondent**: Thank you, I used to experience repetitive falls, so I could not do any work

**Interviewer**: Tell me those things that prompt you to adhere to anti-seizure medications?

**Respondent**: I love my job, and I also fear to be known by others that I get seizures, if I don’t take medications, they are likely to know that I get seizures and may chase me from the work, so I have to take them

**Interviewer**: Are there things that you have to say about anti-seizure medications for the time you have been adhering to it

**Respondent**: The medications work, you still get seizures but the effects are not much

**Interviewer**: How do other people with epilepsy experience adhering to anti-seizure medications?

**Respondent**: I think the medications are good, they like to take them, but sometimes the pressure from the family is high, others even hide these medications sometimes, and also throw away some

**Interviewer**: I would like to know the advantages of adhering to anti-seizure medications, what are they?

**Respondent:** Not to die, to be happy. I like them because I feel better and everything is going on normally, we have seen these medications helping a lot

**Interviewer**: What are the challenges that people who adhere to anti-seizure medications face

**Respondent**: The people who see us are not happy, yes that the medications are not good, that we are always likely to fall any time, that I why I even stop attending gatherings

**Interviewer**: Tell me the community views about adhering to anti-seizure medications

**Respondent**: They are not happy, some people do not respect us, people say that the medications take a lot of time to work yet there are herbals which work very fast to prevent the seizures

**Interviewe**r: To you, how can adhering to anti-seizure medications anti-seizure medications be improved?

**Respondent:** Giving more time to patients, including offering individualized care to taking medications as they are given

**Interviewer**: Tell me the days that you have adhered to your anti-seizure medications in the last five days, did you take them yesterday

**Respondent:** I took my medications all the time, including the days when I travelled. I cannot miss the medications if I have them, but last month I missed because they were not enough

**Interviewe**r: Please tell me the kinds of anti-seizure medications that you know, what are they?

**Respondent:** Three types, all are white and they work for me

**Interviewer**: Ok, would you recommend a person with epilepsy to adhere to medications anti-seizure medications? Why

**Respondent:** Yes I would, because they are good, and work

**Interviewer**: How would you comment on the current available guidelines concerning adherence to anti-seizure medications?

**Respondent:** They are good, so they help us to take the medications

**Interviewer**: Anything else you want to say about anti-seizure medications

**Respondent:** No, that is all

**Interviewer**: Thank you for participating
