# Supplementary material for: Adherence to anti-seizure medications among persons living with epilepsy attending a community- based epilepsy clinic in Buikwe and Mukono Districts-Uganda: A mixed methods study
Source: PLoS One. 2026 Aug 21;21(8):e0356494. doi: 10.1371/journal.pone.0356494 (PMC13496452; doi:10.1371/journal.pone.0356494)
Supplement: Supplementary file 3 [file pone.0356494.s003.zip › IDI 9.docx]

**Participant 9**

**Interviewer**: Next, I would like to know what you understand by adhering to anti-seizure medications.

**Respondent:** It means taking my medications for the disease so as to be well every day, and to be with others.

**Interviewer**: Ok, how did you get to know about adhering to anti-seizure medications?

**Respondent:** I was sent by a friend at the clinic to receive the medications there, and then my mum also guided me, and ensured that I have to go to the clinic daily

**Interviewer**: Then, how did you start adhering to anti-seizure medications?

**Respondent**: When I went to the clinic, I also found my friends there, and the health care workers were also many, so they told me the benefits of the medications. This is what made me start taking these medications.

**Interviewer**: Tell me those things that prompt you to adhere to anti-seizure medications?

**Respondent**: I think I should go back to school, and do my own work so as to earn some money for a living, I feel proud of myself when I work to earn money

**Interviewer**: Are there things that you have to say about anti-seizure medications for the time you have been adhering to it. Do the medications work?

**Respondent:** If you eat well, you feel so happy and healthy with the medications, that is what I can think, but generally they are good to take, no problem sir

**Interviewer**: How do other people with epilepsy experience adhering to anti-seizure medications?

**Respondent**: For sure I don’t know much, but I think they like taking the medications, they say that their relatives fear them if they fail to take the medications.

**Interviewer**: I would like to know the advantages of adhering to anti-seizure medications, what are they?

**Respondent:** Ok, I tell you everyone starts to like staying with you, you are respected, and feel good generally, and you are also like everyone at home

**Interviewer**: What are the challenges that people who adhere to anti-seizure medications face

**Respondent**: Nothing much, but may be sometimes doctors come with few medications, but we find time to discuss with them. May be also sometimes doctors come late

**Interviewer**: Ok, can you tell me the community views about adhering to anti-seizure medications?

**Respondent**: Some views are good, but others worry us, but some community members even help us by giving me some money whenever I am sick, so sometimes their views are good and they are concerned.

**Interviewer**: To you, how can adhering to anti-seizure medications be improved?

**Respondent**: I think taking all the medications with good food may be Ok, that’s Ok, yes like that, I need to be patient, some medications are a sour, and others big to be swallowed

**Interviewer**: Tell me the days that you have adhered to your anti-seizure medications in the last five days, did you take them yesterday

**Respondent**: Daily, I don’t miss my medications, they are good to take thank you, I have to continue taking them, and this is great

**Interviewer**: Ok, great. Please tell me the kinds of anti-seizure medications that you know, what are they?

**Respondent**: Oh, can I bring them and you see, ok, I am not sure but are all so good, three types but I am not sure of their names, but I have taken them for some years

**Interviewer**: Oh, I see. Thank you, can we continue, how would you recommend a person with epilepsy to adhere to medications anti-seizure medications?

**Respondent**: Yes, they should take them all, the medications are good. You know some headache come but you become better, yes

**Interviewer**: How would you comment on the current available guidelines concerning adherence to anti-seizure medications?

**Respondent**: I am not sure, but I think they work, yes they work well especially if taken with water if you take on empty stomach they can make you week, also you need to keep jogging around to make them work well

**Interviewer**: Anything else you want to say about anti-seizure medications

**Respondent:** No. **t**hank you for asking these interesting questions

Interviewer: Thank you for participating
