# Supplementary material for: Adherence to anti-seizure medications among persons living with epilepsy attending a community- based epilepsy clinic in Buikwe and Mukono Districts-Uganda: A mixed methods study
Source: PLoS One. 2026 Aug 21;21(8):e0356494. doi: 10.1371/journal.pone.0356494 (PMC13496452; doi:10.1371/journal.pone.0356494)
Supplement: Supplementary file 3 [file pone.0356494.s003.zip › IDI 10.docx]

**Participant 10**

**Interviewer**: Next, it is always important to know what it means by adhering to anti-seizure medications. I would like to know what you understand by adhering to anti-seizure medications.

**Respondent**: I think taking medications for epilepsy well, as they are given out at the clinic, that is what I think, and also to live longer

**Interviewer**: Ok, and then the next one is to find out how you got to know about adhering to anti-seizure medications. How did you get to know about adhering to anti-seizure medications?

**Respondent**: I got to know from the community health worker, who is responsible for coordinating the epilepsy clinics, he told us that the medications are good, and everyone who takes them can become better, so the next month I also went to the clinic

**Interviewer**: Please, how did you start adhering to anti-seizure medications?

**Respondent**: I saw others at the clinic they were looking good, so I also started to take the drugs like them, and also my relatives also wanted me to continue taking the medications

**Interviewer**: Tell me those things that prompt you to adhere to anti-seizure medications?

**Respondent**: I am interested in working, and may be to also be at home very well, the medications are also interesting, so I feel like continuing with them

**Interviewer**: tell me things that you have to say about anti-seizure medications for the time you have been adhering to it

**Respondent**: It is good to swallow them after meals, they are good medications, for me that is what I think

**Interviewer**: How do other people with epilepsy experience adhering to anti-seizure medications?

**Respondent:** I am not sure, but they don’t say much, but I hear them saying that the medications are good for them, and they work

**Interviewer**: Next is to know the benefits of adhering to anti-seizure medications. What are the advantages of adhering to anti-seizure medications, what are they?

**Respondent**: It is always good to swallow these medications, you start feeling that you are normal, and able to be independent like other normal people, you also feel energetic

**Interviewer**: I know there are challenges associated with adherence to anti-seizure medications. What are the challenges that people who adhere to anti-seizure medications face

**Respondent**: Those medications work, but we walk long distances to the clinic, sometime we walk these long distances when we are hungry, and yet we get seizures

**Interviewer**: I know even the community has views associated with anti-seizure medications. Tell me the community views about adhering to anti-seizure medications

**Respondent:** I am not sure, like I said before, but for me I feel healthy, they say many things like the medications do not work, but they also like giving me food sometimes when they have, ok, so maybe they have different views

**Interviewer**: To you, how can adhering to anti-seizure medications be improved?

**Respondent:** Working as a team to clinic and may be to give us the medications any time we need them, or keeping time, ok, that is what I think

**Interviewer**: Tell me the days that you have adhered to your anti-seizure medications in the last five days, did you take them yesterday

**Respondent**: I take every day, to tell you I like them, they are very good for me, isn’t it, what do you say please, isn’t that fine

**Interviewer**: Oh, I believe it is fine. Please tell me the kinds of anti-seizure medications that you take, what are they?

**Respondent**: Tegretol, and three other types, which I take daily, and they are always given out, I have to rest after taking. Some are Capsules, and others are tablets of different sizes

**Interviewer**: How would you recommend a person with epilepsy to adhere to medications anti-seizure medications?

**Respondent**: If they need to be healthy, they should take their medications, they are good and available, and sometimes all the medications are there.

**Interviewer**: How would you comment on the current available guidelines concerning adherence to anti-seizure medications?

**Respondent:** I am not sure of the available guidelines, you mean knowing how to use the medications; they are good, doctors communicate well, are available and ready to corporate, thanks great.

**Interviewer**: Anything else you want to say about anti-seizure medications

**Respondent**: May be all has been discussed, nothing else

**Interviewer**: Thank you for participating
