# Supplementary material for: Adherence to anti-seizure medications among persons living with epilepsy attending a community- based epilepsy clinic in Buikwe and Mukono Districts-Uganda: A mixed methods study
Source: PLoS One. 2026 Aug 21;21(8):e0356494. doi: 10.1371/journal.pone.0356494 (PMC13496452; doi:10.1371/journal.pone.0356494)
Supplement: Supplementary file 3 [file pone.0356494.s003.zip › IDI 11.docx]

**Participant 11**

**Interviewer**: I would like to know what you understand by adhering to anti-seizure medications.

**Respondent:** It means taking my anti-seizure medications as being given by the health care providers, including eating on time and having adequate meals and also respecting others who take drugs. Also taking drugs on time such that you can have energy

**Interviewer:** How did you get to know about adhering to anti-seizure medications?

**Respondent:** I was motivated by patients with epilepsy in our community who were also taking the medications, I also used to see people going to the clinic, so I thought following them was a good idea

**Interviewer**: please, how did you start adhering to anti-seizure medications?

**Respondent:** My mum asked me to do so, and everybody at home, initially I was using local herbs which were not working very well. They say I used to get frequent seizures many times a day, so they also asked me to start using anti-seizure medications

**Interviewer**: Tell me those things that prompt you to adhere to anti-seizure medications?

**Respondent**: I had suicidal thoughts, I wanted to harm myself, so everybody at home was very strict on me, to avoid these bad thoughts, I had to take the medications, I also never wanted others to fear me.

**Interviewer**: Tell me things that you have to say about anti-seizure medications for the time you have been adhering to it

**Respondent**: I now have good thoughts and good health, I am feeling better than I was, I tell you sir the anti-seizure medications really work, and they are good

**Interviewer**: How do other people with epilepsy experience adhering to anti-seizure medications?

**Respondent:** They sometimes tell me to take the medications, while others do not want to use them, I don’t know why, but for me I like them, some patients take these medications, and I encourage them to continue taking them for better health.

**Interviewer**: I would like to know the advantages of adhering to anti-seizure medications, what are they?

**Respondent**: Thank you, the most important is that you reduce on the number of seizures, and can have good health like me, I have to tell you the medications benefit me

**Interviewer**: What are the challenges that people who adhere to anti-seizure medications face

**Respondent:** Oh, yes, long distance and failure to wait for doctors at the clinic due to lack of food, also the medications can bore, I think something should be done, ok. Like for me every day I take 3 types of drugs two times per day for now four years and have always been taking them as instructed by health care providers, however the moment I stop taking any of them, I get an attack, this worries me a lot

**Interviewer**: Tell me the community views about adhering to anti-seizure medications

**Respondent:** They sometime give some support like money to go to the clinic, but not always, others may provide free ride to the clinic sometimes, some community members are concerned and see us as patients but others not. Even sometimes they give me food if they like, so I cannot blame them.

**Interviewer**: To you, how can adhering to anti-seizure medications anti-seizure medications be improved?

**Respondent**: Let us continue taking the medications well, they can work; other people are now better after taking them, we need to continue taking them, there is need for more counseling to patients

**Interviewer**: Ok, can you tell me the days that you have adhered to your anti-seizure medications in the last five days

**Respondent:** I took my anti-seizure medications all the days, only that they are not enough for this week, I have been taking them all the days, ok, I cannot miss any of them

**Interviewer**: Please tell me the kinds of anti-seizure medications that you know, what are they?

**Respondent**: I don’t know their names but sometimes they are changed them, I now take 3 types, which are good but they make me weak most especially immediately after swallowing, therefore I have to drink a lot of water

**Interviewer**: There I always a need to recommend a patient to take medications, how would you recommend a person with epilepsy to adhere to medications anti-seizure medications?

**Respondent**: Let they use the anti-seizure medications very well, counseling on how to use the medications is also very important so as to be healthy, community members should also be very supportive to patients living with epilepsy, I think, but otherwise, the medications are good if used well

**Interviewer**: How would you comment on the current available guidelines concerning adherence to anti-seizure medications?

**Respondent**: They guide as well, I think they are good guides, let us continue following them, we need to use the medications very consistently

**Interviewer**: That is all for this interview, thank you for participating
