# Supplementary material for: Adherence to anti-seizure medications among persons living with epilepsy attending a community- based epilepsy clinic in Buikwe and Mukono Districts-Uganda: A mixed methods study
Source: PLoS One. 2026 Aug 21;21(8):e0356494. doi: 10.1371/journal.pone.0356494 (PMC13496452; doi:10.1371/journal.pone.0356494)
Supplement: Supplementary file 3 [file pone.0356494.s003.zip › IDI 12.docx]

**Participant 12**

**Interviewer**: Next, I would like to know what you understand by adhering to anti-seizure medications.

**Respondent**: It means taking medications on time after eating food every day, so as to avoid seizures, this is what I was told to do by the doctors.

**Interviewer**: How did you get to know about adhering to anti-seizure medications?

**Respondent**: I saw people with epilepsy going to the clinic, and followed them,also one patient came for me, so my parents also asked me to take the medications, I like to do so myself, I ensure that all my medications availed to me every month by ensuring that I am present at the clinic

**Interviewer**: Please, how did you start adhering to anti-seizure medications?

**Respondent**: Like I have mentioned, I used to see patients going to the clinic, but again I saw them taking the medications, and I also picked interest in taking them

**Interviewer**: Tell me those things that prompt you to adhere to anti-seizure medications?

**Respondent**: The key thing is the need for my health sir, I have to take these medications well so as to be healthy, they are good medications. I also never wanted to be discriminated from the community gatherings. Oh, they are good thanks for that question

**Interviewer**: Our next question is, are there things that you have to say about anti-seizure medications for the time you have been adhering to them

**Respondent**: They are heavy medications especially when taken on empty stomach, it needs to be swallowed with water sir, some make you sleep a lot, and also difficult to perform basic work related activities, which is a challenge for a man please. I therefore have to eat a lot

**Interviewer**: How do other people with epilepsy experience adhering to anti-seizure medications?

**Respondent:** Others are not responsible; however others are responsible, yes, they like the medications sometimes, I think people need support with food and money to take the medications, it is difficult to use them well I tell you

**Interviewer**: So sensitization is needed, next I would like to know the advantages of adhering to anti-seizure medications, what are they?

**Respondent:** First the seizures reduce greatly, but with good meals, second you can do some income generating activity for the family, I believe that can also better than nothing, we should be happy always, I think

**Interviewer**: What are the challenges that people who adhere to anti-seizure medications face

**Respondent**: Oh, may be long distance travel, we generally move on foot, making it difficult to the clinic on time, sometimes we buy medications if are out of stock, some medications are big in size, so difficult to maintain them sometime please

**Interviewer**: Tell me the community views about adhering to anti-seizure medications

**Respondent**: We are supported sometimes, others say we are not supposed to attend traditional rituals; some even hate seeing some medications. But they support us reach the clinic if we are not having money

**Interviewer**: To you, how can adhering to anti-seizure medications be improved?

**Respondent**: I think more community sensitization is needed, especially in villages, also may be they can give even more and more medications for at least three months, then we can save time to go there every month. I think that would be good

**Interviewer**: Tell me the days that you have adhered to your anti-seizure medications in the last five days, did you take them yesterday

**Respondent**: I always do so everyday please, I don’t miss but sometimes I feel tired sir, they irritate sometimes hence difficult to swallow

**Interviewer**: Ok, great. Please tell me the kinds of anti-seizure medications that you know, what are they?

**Respondent**: Capsules and tablets, four capsules per day and two white tablets, they even work for me I tell you sir, they changed them sometime about three months back but I did not feel well, as I started over sleeping

**Interviewer**: How would you recommend a person with epilepsy to adhere to medications anti-seizure medications?

**Respondent**: We need counseling to take the medications, let us not relax please, I believe that they may work for us, may be also to be given a lot of food

Interviewer: How would you comment on the current available guidelines concerning adherence to anti-seizure medications?

**Respondent:** They are ok, let us continue taking the anti-seizure medications

**Interviewer**: That is the end of the interview thank you very for participating
