# Supplementary material for: Adherence to anti-seizure medications among persons living with epilepsy attending a community- based epilepsy clinic in Buikwe and Mukono Districts-Uganda: A mixed methods study
Source: PLoS One. 2026 Aug 21;21(8):e0356494. doi: 10.1371/journal.pone.0356494 (PMC13496452; doi:10.1371/journal.pone.0356494)
Supplement: Supplementary file 3 [file pone.0356494.s003.zip › IDI 13.docx]

**Participant 13**

**Interviewer**: I what do you understand by adhering to anti-seizure medications.

**Respondent**: I think taking medications on time, but you must first eat to take them as they are strong, that is what I think

**Interviewer**: Then, how did you get to know about adhering to anti-seizure medications?

**Respondent**: Thank you, I got to know from my parents, they took me to the clinic to receive anti-seizure medications, and started treatment; they were also confused about the falls

**Interviewer**: Ok, please, how did you start adhering to anti-seizure medications?

**Respondent**: My parents ensured that I take the medications, they also saw others going to the clinic, and they also wanted me to take

**Interviewer**: Please tell me those things that prompt you to adhere to anti-seizure medications?

**Respondent**: For me I am a working person, who needed to earn a living I tell you. If I don’t take medications I may be chased from work, for example, when I was taken to Kampala to porter on a building…, my boss got to know that I get epileptic seizures, they asked me to go back home, this led me to ensure that I take my medication not to get epileptic seizures

**Interviewer**: Are there things that you have to say about anti-seizure medications for the time you have been adhering to it

**Respondent**: Not taking them make me lose concentration, and become very abusive, so I think they are good medications to take, I would become a burden to others I tell you

**Interviewer**: How do other people with epilepsy experience adhering to anti-seizure medications?

**Respondent**: I think those who don’t use are difficult to control, some have wounds, but those who use the medications are healthy and energetic, may be, that is what I think

**Interviewer**: I would like to know the advantages of adhering to anti-seizure medications, what are they?

**Respondent**: Making us happy and confident, family members don’t run away from you, you can even work, so I like them

**Interviewer**: What are the challenges that people who adhere to anti-seizure medications face

**Respondent**: Sometimes the medications are few, requires to be kept well, there are days I receive medications for 15 days instead of 30 days like required. There is difficult to find what to eat on time especially if you are not working

**Interviewer**: On the side of the community, tell me the community views about adhering to anti-seizure medications

**Respondent**: Yes, they are helpful, but some try to stop us from taking drugs, a big challenge, they think we were sacrificed which is not correct at all, others say we cannot do anything for a leaving

**Interviewer**: To you, how can adhering to anti-seizure medications be improved?

**Respondent:** Supervising the patients, those not taking the medications well should be reported. I think we have to be helped

**Interviewe**r: Tell me the days that you have adhered to your anti-seizure medications in the last five days, did you take them yesterday

**Respondent**: I last took yesterday; they are good so I liked them. I try to take every day as I can

**Interviewer**: Al right, please tell me the kinds of anti-seizure medications that you take, what are they?

**Respondent**: I take two types, all capsules, I like them and believe they are working, can’t tell their types, but I know them all, they have changed my life please

**Interviewer**: How would you recommend a person with epilepsy to adhere to medications anti-seizure medications?

**Respondent**: Let they take the medications well, as they prevent poor thoughts

**Interviewer**: Do you think the current guidelines concerning adherence to anti-seizure medications are good, comment on the current available guidelines concerning adherence to anti-seizure medications?

**Respondent:** They work, seizures are controlled and everything can be well

**Interviewer**: That is all, **t**hank you for participating in this interview
