# Supplementary material for: Adherence to anti-seizure medications among persons living with epilepsy attending a community- based epilepsy clinic in Buikwe and Mukono Districts-Uganda: A mixed methods study
Source: PLoS One. 2026 Aug 21;21(8):e0356494. doi: 10.1371/journal.pone.0356494 (PMC13496452; doi:10.1371/journal.pone.0356494)
Supplement: Supplementary file 3 [file pone.0356494.s003.zip › IDI 14.docx]

**Participant 14**

**Interviewer**: Please, what do you understand by adhering to anti-seizure medications.

**Respondent**: Taking all my anti-seizure medications well as given to me by the doctors

Interviewer: Then, how did you get to know about adhering to anti-seizure medications?

**Respondent**: For very many years, I have been taking my medications; I wanted them, since I needed to save life, as to be like other people who are ok

**Interviewer**: please, how did you start adhering to anti-seizure medications?

**Respondent**: I needed to support my family, all the children need food so I needed to work for them, and the doctors are also my friends

**Interviewer**: Tell me those things that prompt you to adhere to anti-seizure medications?

**Respondent**: To have good health so as to be able to work, that is what I think for now, may be. I also need my health I tell you, ok

**Interviewer**: tell me things that you have to say about anti-seizure medications for the time you have been adhering to it

**Respondent**: The medications are good, these also prevent worries and fears I think, always let us take them well I can say

**Interviewer**: How do other people with epilepsy experience adhering to anti-seizure medications?

**Respondent**: It depends, for others the medications can cure epilepsy, but maybe I don’t think, I know many people who have been with the disease for long time

**Interviewer**: I would like to know the advantages of adhering to anti-seizure medications, what are they?

**Respondent**: You feel healthy; your people see you as important, also you can be cured of epilepsy. I get reduced pain when I consistently use the medications

**Interviewer**: What are the challenges that people who adhere to anti-seizure medications face

**Respondent**: May be too long distance to the clinic as others come from very far, over 8kms yet some have to walk on foot. Some think medications don’t work and use herbals which may or may not work

**Interviewer**: Tell me the community views about adhering to anti-seizure medications

**Respondent**: It depends, others are supportive while some don’t see any advantage of using these medications, and they think people taking anti-seizure medications also have other chronic conditions which have failed to heal

**Interviewer**: To you, how can adhering to anti-seizure medications anti-seizure medications be improved?

**Respondent:** I think, mostly liking the medications, also eating well, that is what I think. You know these medications are strong to be swallowed daily. There is need of bringing more medications to the clinic

**Interviewer**: Ok, then tell me the days that you have adhered to your anti-seizure medications in the last five days, did you take them yesterday

Respondent: I have followed all the days well, even today the medications are available, I only missed a few days last month

**Interviewer**: Please tell me the kinds of anti-seizure medications that you know, what are they?

Respondent**:** Three types, capsules and tablets, taken daily. In the morning two tablets and two capsules

**Interviewer**: How would you recommend a person with epilepsy to adhere to medications anti-seizure medications?

**Respondent**: They should take the medications well as given, they really work

**Interviewer**: How would you comment on the current available guidelines concerning adherence to anti-seizure medications?

**Respondent**: I am not sure of the current guidelines, but we are told to use the medications well

**Interviewer**: Anything else you want to say about anti-seizure medications

**Respondent**: No, we have discussed all

**Interviewer**: Ok, thank you for participating in the interview
