# Supplementary material for: Adherence to anti-seizure medications among persons living with epilepsy attending a community- based epilepsy clinic in Buikwe and Mukono Districts-Uganda: A mixed methods study
Source: PLoS One. 2026 Aug 21;21(8):e0356494. doi: 10.1371/journal.pone.0356494 (PMC13496452; doi:10.1371/journal.pone.0356494)
Supplement: Supplementary file 3 [file pone.0356494.s003.zip › IDI 15.docx]

**Participant 15**

**Interviewer**: I would like to know what you understand by adhering to anti-seizure medications.

**Respondent**: Following instructions while using anti-seizure medications, they are the ones we use, as given by our doctors

**Interviewer**: How did you get to know about adhering to anti-seizure medications?

**Respondent**: They were given to me by the doctors, I was checked and given the medications later, they are working, and also my husband is responsible so he took me there to receive the treatment

**Interviewer**: Looking at the initial stages before using the medications, how did you start adhering to anti-seizure medications?

**Respondent:** I was advised by the doctors, they asked me to take so as to avoid seizures. I realized we had a family problem, hence had to take the medications to be healed, yes, oh, that is how I started, and I have benefited somehow from the medications

**Interviewer**: Next, what are the prompts that enable you to adhere to anti-seizure medications?

**Respondent**: The family is supportive; I also need to do my business, and expanding on it so when I use my medications well, I feel strong and motivated everyday

**Interviewer**: Are there things that you have to say about anti-seizure medications for the time you have been adhering to it, do the medications work?

**Respondent**: They work but difficult to access if you miss them, also require a lot of eating food, they help to relieve discomfort and pain, yes pain my body pains a lot without medications, because I fall frequently and get injuries, so the medications reduce the falls

**Interviewer**: How do other people with epilepsy experience adhering to anti-seizure medications? How do they view other people with epilepsy?

**Respondent**: I am not sure but may be they are ok, I see them saying the medications work for them whenever they come to the clinic, sometimes I see them helping out others especially those who are new, so I think the medications work, ok

**Interviewer**: I would like to know the advantages of adhering to anti-seizure medications, what are they?

**Respondent**: Like I said, you feel healthy and strong, you also feel cured and treated of the disease.

**Interviewer**: What are the challenges that people who adhere to anti-seizure medications face

**Respondent**: The costs, especially of sodium valporoate, this is very expensive to buy in case you miss it at the clinic, however the medications that I receive these days are at least cheap to buy, Tegretal and folic acid, however if you are poor you may not manage, let me tell you

**Interviewer**: Tell me the community views about adhering to anti-seizure medications

**Respondent:** It depends on the community, they say epilepsy is inherited and affects those with HIV, so one has to use medications for both HIV and epilepsy which sometimes challenge

**Interviewer**: To you, how can adhering to anti-seizure medications be improved?

**Respondent**: Reducing on the costs and bringing more medications to patients may help, we need to be given more foods when at home, also the doctors need to start the clinic early, other clients dose at the clinic while waiting and get seizures

**Interviewer**: Tell me the days that you have adhered to your anti-seizure medications in the last five days, did you take them yesterday

**Respondent**: I have no problem, I take all the drugs daily, sometimes the husband supports me, and also the children help pick the medications from the facility whenever I am for work, so I can’t miss any of my dosages, so I have taken the medications well in the last five days

**Interviewer**: Please tell me the kinds of anti-seizure medications that you know, what are they?

**Respondent:** Tegretal and folic acid are what I take; they are good medications for me. Every day I like to take these medications well

**Interviewer**: Would you recommend a person with epilepsy to adhere to medications anti-seizure medications?

**Respondent**: Well, I can recommend them, the medications can work if one is seriously taking them, that’s it but they should keep using them

**Interviewer**: How would you comment on the current available guidelines concerning adherence to anti-seizure medications?

**Respondent**: I am not sure, you mean the number of health workers?, they are enough but may be not coming early is still a problem, the number of patients are increasing everyday so the doctors also have to increase, even the speed is low, we come from very far so we get tired

**Interviewer**: Anything else you want to say about anti-seizure medications

**Respondent: No**

**Interviewer**: Thank you for the responses
