# Supplementary material for: Adherence to anti-seizure medications among persons living with epilepsy attending a community- based epilepsy clinic in Buikwe and Mukono Districts-Uganda: A mixed methods study
Source: PLoS One. 2026 Aug 21;21(8):e0356494. doi: 10.1371/journal.pone.0356494 (PMC13496452; doi:10.1371/journal.pone.0356494)
Supplement: Supplementary file 3 [file pone.0356494.s003.zip › IDI 16.docx]

**Participant 16**

**Interviewer**: First, what you understand by adhering to anti-seizure medications.

**Respondent**: Taking medications for epilepsy as expected, or taking them well at the right time of the day, may be also to take all of them

**Interviewer**: How did you get to know about adhering to anti-seizure medications?

**Respondent**: A community health worker came to our home to inform us about the epilepsy clinic, he is always very so I had to use the medications

**Interviewer**: Ok, then how did you start adhering to anti-seizure medications?

**Respondent**: I believe the medications are good, at one time a friend asked me to be using the medications, that I was not behaving well, I used to injure myself, I would also fall in fire at one time, even the doctors were very clear that I should take the medications, so I started taking them well

**Interviewer**: Tell me those things that prompt you to adhere to anti-seizure medications?

**Respondent**: I like to be in public being a teacher, so people should not get to know that I have epilepsy, not taking them may make people aware that I have epilepsy, so I need to take my medications please

**Interviewer**: For the time you have been taking these medications as recommended, tell me things that you have to say about anti-seizure medications, do they work

**Respondent**: They cause heart diseases, we have to be cautious when taking them as they can be a problem most especially if used for very many years, but I have seen them working, actually even the family members say that

**Interviewer**: How do other people with epilepsy experience adhering to anti-seizure medications?

**Respondent**: They like the medications, there is no alternative for them, others need to consistently be reminded about the medications they are taking, forgetting to take the medications exposes you to burns, you can burn your leg while cooking, others say the medications cause infertility and increased sexual moods especially the men

**Interviewer**: Then, I would like to know the advantages of adhering to anti-seizure medications, what are they?

**Respondent**: You can have improved quality of life, start doing basic home activities for yourself and the family, sometimes also being liked by the community

**Interviewe**r: What are the challenges that people who adhere to anti-seizure medications face

**Respondent**: Over walking, you also easily become dizzy after taking the medications sometimes also fatigue and loss of memory, also being given the same type of medications every year is very troublesome

**Interviewer**: Tell me the community views about adhering to anti-seizure medications

**Respondent**: People say that people living with epilepsy are already bewitched and anti-seizure medications cannot work, they are believed to have wronged others in communities, and not fit to be leaders, the medications are believed to affect the internal organs

**Interviewer**: To you, how can adhering to anti-seizure medications anti-seizure medications be improved?

**Respondent**: May be providing employment opportunities to people living with epilepsy, people have to be employed to look for transport facilitation to the clinic, I guess that can help please, also health care providers should give out the medications to patients as they are instructed, that may help

**Interviewer**: Tell me the days that you have adhered to your anti-seizure medications in the last five days, did you take them yesterday

**Respondent**: Every day I take my medications. I only missed once when I had travelled, when I am not given all medications, I have to buy them from the pharmacy

**Interviewer:** Please tell me the kinds of anti-seizure medications that you know, what are they?

**Respondent:** I take Lamotrigene, Tegretal and Folic acid, all are big drugs in size except folic acid, but they all work well

**Interviewer**: How would you recommend a person with epilepsy to adhere to medications anti-seizure medications?

**Respondent**: Just take the medications well. They can only live well like others if they take their medication but also to always be active in work, they need to eat a lot of food

**Interviewer**: How would you comment on the current available guidelines concerning adherence to anti-seizure medications?

**Respondent**: They are good but more is still needed, imagine waiting for 3 hours before receiving medications

**Interviewer**: Anything else you want to say about adherence anti-seizure medications

**Respondent**: No

**Interviewer**: Thank you for your responses
