# Supplementary material for: Adherence to anti-seizure medications among persons living with epilepsy attending a community- based epilepsy clinic in Buikwe and Mukono Districts-Uganda: A mixed methods study
Source: PLoS One. 2026 Aug 21;21(8):e0356494. doi: 10.1371/journal.pone.0356494 (PMC13496452; doi:10.1371/journal.pone.0356494)
Supplement: Supplementary file 3 [file pone.0356494.s003.zip › IDI 17.docx]

**Participant 17**

**Interviewer**: Once again thank you for accepting to participate in this interview. First, I would like to know what you understand by adhering to anti-seizure medications.

**Respondent**: This may mean taking all your medications I think, they always tell us to do that, with good food, also following all the instructions concerning taking all the medications

**Interviewer**: Then the next question is; how did you get to know about adhering to anti-seizure medications?

**Respondent**: I was told by my family members, also the doctors that I have to take all the medications well

**Interviewer**: please, how did you start adhering to anti-seizure medications?

**Respondent**: Because I used to get seizures, my parents started buying some medications for me but later I was taken to the hospital in Mukono to start getting, that is how I started

**Interviewer**: Tell me those things that prompt you to adhere to anti-seizure medications?

**Respondent**: I need free life always, I also need to be comfortable, so I have to take the medications, may be also to be healthy

**Interviewer**: tell me things that you have to say about anti-seizure medications for the time you have been adhering to it, do these medications work

**Respondent**: They are good drugs, also the period I have been using them is so long, more than 10 years, but they really work for me

**Interviewer**: How do other people with epilepsy experience adhering to anti-seizure medications?

**Respondent**: The medications are good, patients that have used them for many years keep encouraging us to keep using them, they say that one can recover from epilepsy when uses the medications very well

**Interviewer**: What are the advantages of adhering to anti-seizure medications, what are they?

**Respondent**: You become more confident, able to do your own work with minimal support, and also they relieve stress if you drink a lot of water

**Interviewer**: What are the challenges that people who adhere to anti-seizure medications face

**Respondent:** Walking long distances, and not having some money, but the doctors are always there to work on us, also sometimes headache and increased appetite, you have to eat a lot yet food is not there

**Interviewer**: Tell me the community views about adhering to anti-seizure medications

**Respondent**: They say that the condition is inherited, so chances of recovery are minimal, but we just have to take the medications well, maybe they can work, however if I am unable to work they support me*,* If my people are in the garden, the neighbors always give me food such that I can take my medications on time

**Interviewer**: To you, how can adhering to anti-seizure medications be improved?

**Respondent**: Taking all the medications well, I think that is the best, may be also nearby anti-seizure distribution points be started, that may work, I also think people should like us

**Interviewer**: Tell me the days that you have adhered to your anti-seizure medications in the last five days, did you take them yesterday

**Respondent**: Yes I took my drugs yesterday and the other day, maybe I missed once, they are getting finished so I fear finishing them before the month ends

**Interviewer**: Please tell me the kinds of anti-seizure medications that you know, what are they?

**Respondent**: Two types, Tegretol and folic acid, they have worked for me, but sometime they also add another medication which I don’t know

**Interviewer**: How would you recommend a person with epilepsy to adhere to medications anti-seizure medications?

**Respondent**: We just have to use them well, let’s not be bored as they work

**Interviewer**: How would you comment on the current available guidelines concerning adherence to anti-seizure medications?

**Respondent**: They are good but something should be changed, you see some medications are very expensive if you miss them, can they increase on the number of clinic days, I think that may also work for the patients

**Interviewer**: That is the end, Thank you for participating in this interview
