# Supplementary material for: Adherence to anti-seizure medications among persons living with epilepsy attending a community- based epilepsy clinic in Buikwe and Mukono Districts-Uganda: A mixed methods study
Source: PLoS One. 2026 Aug 21;21(8):e0356494. doi: 10.1371/journal.pone.0356494 (PMC13496452; doi:10.1371/journal.pone.0356494)
Supplement: Supplementary file 3 [file pone.0356494.s003.zip › IDI 18.docx]

**Participant 18**

**Interviewer**: I would like to know what you understand by adhering to anti-seizure medications.

**Respondent**: Taking all medications well, I think that is what it means to me

**Interviewer**: How did you get to know about adhering to anti-seizure medications?

**Respondent**: I have always been taking, since my mother was informed about the benefits, she tells me that the medications are good, and even the doctors say that

**Interviewer**: Please, how did you start adhering to anti-seizure medications?

**Respondent:** I think mum was told that they are good medications, so I have been taking them for many years and they work

**Interviewer**: Tell me those things that prompt you to adhere to anti-seizure medications? Do the anti-seizure medications work?

**Respondent**: Yes they work, I do not want to bother others, they are overwhelmed of the condition especially when I get a seizure; I also need to be healthy to enjoy life please

**Interviewer**: tell me things that you have to say about anti-seizure medications for the time you have been adhering to it

**Respondent**: They give headache sometimes, especially if you take them without food, you have to eat a lot of food, oh yes.

**Interviewer**: How do other people with epilepsy experience adhering to anti-seizure medications?

**Respondent**: The medications make them sleep, others just give up taking medications, even the number of patients at the clinic is not consistent, some of say that the medications sometimes do not work well for them

**Interviewer**: Ok, I would like to know the advantages of adhering to anti-seizure medications, what are they?

**Respondent**: Mostly is being able to do your own work without limitations, for example you become part of the group, you are not isolated, I like being with others, and enjoy good life

**Interviewe**r: What are the challenges that people who adhere to anti-seizure medications face

**Respondent**: Some parents don’t know the benefits of medications, so they need to be informed always, they end up also not telling their children to take the medications, so parents lack proper information about the anti-seizure medication

**Interviewer**: Tell me the community views about adhering to anti-seizure medications

**Respondent**: They say we were sacrificed, our parents wanted to get rich so they sacrificed us, it is so challenging but nothing to do, however some other people are concerned, they help us with looking for water and provide food sometimes, many have seen us good people

**Interviewer**: How can adhering to anti-seizure medications anti-seizure medications be improved?

**Respondent**: Just using them as being told can help, also taking the right dosage, listening to the doctors, may be to just take the given medications well, we should not give up taking them

**Interviewer**: Tell me the days that you have adhered to your anti-seizure medications in the last five days, did you take them yesterday

**Respondent**: I always take the medications every day, I cannot miss, when I travel I also have to move with them in my bag, everyone at home knows, but I am pregnant, and I think when I take these medications.., I feel they will affect my baby, so, I have to wait taking them until I deliver

**Interviewer**: Please tell me the kinds of anti-seizure medications that you know, what are they?

**Respondent**: Three types, have white and yellow but they all work, I always enjoy taking my medications daily

**Interviewer**: How would you recommend a person with epilepsy to adhere to medications anti-seizure medications?

**Respondent:** They should just take the medications well, as being given, they really work

**Interviewer**: How would you comment on the current available guidelines concerning adherence to anti-seizure medications?

**Respondent**: I think we should just follow them; we need to be supported with food especially when we wait at the clinics for many hours

**Interviewer**: Anything else you want to say about anti-seizure medications

**Respondent**: I think that is what I can say, thanks
