# Supplementary material for: Adherence to anti-seizure medications among persons living with epilepsy attending a community- based epilepsy clinic in Buikwe and Mukono Districts-Uganda: A mixed methods study
Source: PLoS One. 2026 Aug 21;21(8):e0356494. doi: 10.1371/journal.pone.0356494 (PMC13496452; doi:10.1371/journal.pone.0356494)
Supplement: Supplementary file 3 [file pone.0356494.s003.zip › IDI 19.docx]

**Participant 19**

**Interviewer**: Next, kindly I would like to know what you understand by adhering to anti-seizure medications.

**Respondent**: It means having the medications and being healthy

**Interviewer**: Then, how did you get to know about adhering to anti-seizure medications?

**Respondent**: My parents inquired, they were then directed where to seek treatment from. I think that is why they took me to the clinic

**Interviewer**: Please, how did you start adhering to anti-seizure medications?

**Respondent**: My parents actually ensured that I started using the medications the very day I went to the clinic; actually I like the medications because they work, my parents also continue to encourage me to use them

**Interviewer**: Tell me those things that prompt you to adhere to anti-seizure medications?

**Respondent**: To be happy, and behave like anybody else, that’s what I want, the medications can help me to prevent the wounds

**Interviewer**: tell me things that you have to say about anti-seizure medications for the time you have been adhering to it

**Respondent**: They work, seizures do not come again when you take the medications, you feel more relaxed with them, even you become active in daily activities, and no seizure can easily weaken you

**Interviewer**: I am aware there are other people living with epilepsy who also attend the clinic, how do other people experience adhering to anti-seizure medications?

**Respondent:** I cannot tell, but may be they are also feeling better like me, as the medications really work well, some however say they may become infertile, so they are right I may say

**Interviewer**: I would like to know the advantages of adhering to anti-seizure medications, what are they?

**Respondent**: The medications reduce the seizures from 3 to 1 seizure per day, and also to be able to do something for you, yes, that is why I like them

**Interviewer**: What are the challenges that people who adhere to anti-seizure medications face

**Respondent**: Some don’t know the benefits, but also if you get fever the number of attacks increases, like for me I had attacks last week because I had fever, may be the fever also affect the working of the medications

**Interviewer**: Tell me the community views about adhering to anti-seizure medications

**Respondent**: Many things are said, some say epilepsy can be cured by the medications, while others no, sometimes you have to hide the medications from them since they may want to start keeping away from you, the medications are believed to be for those people with mental health challenges, so we have to be very careful when using them, others say we don’t know what we want

**Interviewer**: Ok, thank you for raising that concern. How can adhering to anti-seizure medications anti-seizure medications be improved?

**Respondent:** I think regular attending to the clinic and avoid what other people may say

**Interviewer**: Tell me the days that you have adhered to your anti-seizure medications in the last five days, did you take them yesterday

**Respondent**: Ok, for one or two days I missed the medications, I didn’t forget but just didn’t want to take them, so yes it was a mistake but you know they are difficult to swallow sometime

**Interviewer**: Please tell me the kinds of anti-seizure medications that you know, what are they?

**Respondent:** They are only three types, capsule and tablets, but another medication is provided if one medication is out of stock

**Interviewer**: How would you recommend a person with epilepsy to adhere to medications anti-seizure medications?

**Respondent**: Let them use the medications well, they should always inform the doctors if they get any side effect from the medications

**Interviewer**: How would you comment on the current available guidelines concerning adherence to anti-seizure medications?

**Respondent**: I don’t have problems with them, may be more time is needed to sensitize patients, oh even visiting us at homes

**Interviewer**: Anything else you want to say about anti-seizure medications

**Respondent**: Thanks for the interview

**Interviewer**: Thank you for participating
