# Supplementary material for: Adherence to anti-seizure medications among persons living with epilepsy attending a community- based epilepsy clinic in Buikwe and Mukono Districts-Uganda: A mixed methods study
Source: PLoS One. 2026 Aug 21;21(8):e0356494. doi: 10.1371/journal.pone.0356494 (PMC13496452; doi:10.1371/journal.pone.0356494)
Supplement: Supplementary file 3 [file pone.0356494.s003.zip › IDI 20.docx]

**Participant 20**

**Interviewer**: Thank you very much once again. I would like to know what you understand by adhering to anti-seizure medications.

**Respondent**: It means following the advice of health care workers regarding the use of medications in the way they are given like eating food on time, that’s what I can say, also following the right dosage and time

**Interviewer**: How did you get to know about adhering to anti-seizure medications?

**Respondent**: I was encouraged by the family members to take the medications, so as to be important in the community

**Interviewer**: Ok, please, how did you start adhering to anti-seizure medications?

**Respondent**: They were the parents mainly who wanted to see me taking the medicatios every day, this is how I started using them, I also see people coming for them at the clinic, even the children come for them

**Interviewer**: Tell me those things that prompt you to adhere to anti-seizure medications?

**Respondent**: I have to work to earn a living, this will help me get some money, so I have to use them the way I am told, I think that is why I take the medications. Also to reduce the brain attacks and have improved brain function

**Interviewer**: tell me things that you have to say about anti-seizure medications for the time you have been adhering to it, do the medications work

**Respondent**: The medications work for those who are serious and also those interested in taking the medications… for me I don’t have any problem with them; if I take after eating they are ok but difficult with empty stomach, causing dizziness and weakness sometimes

**Interviewer**: How do other people with epilepsy experience adhering to anti-seizure medications?

**Respondent**: Some people say the medications work, others say they don’t work because they only take them when they want

**Interviewer**: What are the advantages of adhering to anti-seizure medications, what are they?

**Respondent**: The medications reduce the strength of the illness, you may not get the falls if you take them, this is what I always experience

**Interviewer**: What are the challenges that people who adhere to anti-seizure medications face

**Respondent**: Taking too many medications challenges a lot, they are actually difficult to swallow I can tell you, also some people do not have the money to buy all the medications that are not provided, most people thus end up not using them

**Interviewer**: Tell me the community views about adhering to anti-seizure medications

**Respondent**: The communities say the disease can best be treated using local herbs not modern medications, and epilepsy moves in families. So modern medications cannot cure the disease, people should use herbals, that is what people say

**Interviewer**: Ok, then, how can adhering to anti-seizure medications be improved?

**Respondent**: We need to take all the medications well I think, also reducing the medication prices can help, also being consistent in attending to the clinic may help us, I also think that community visits should be carried out by the doctors to encourage patients to take their medications

**Interviewer**: Tell me the days that you have adhered to your anti-seizure medications in the last five days, did you take them yesterday

**Respondent**: I have taken the medications all the days of the week, even last week I took, but sometimes at the end of the month we miss some medications, I think what they provide is not enough for a month

**Interviewer**: Please tell me the kinds of anti-seizure medications that you know, what are they?

**Respondent**: I know Lamotrigine, other drugs I don’t know, but I take three types of medications now for two months, and they have not been changed, sometimes the medications are changed but they have not changed for me for the last two months

**Interviewer**: How would you recommend a person with epilepsy to adhere to medications anti-seizure medications?

**Respondent**: Always not to miss the medications, they should continuously take all of them as given not to have falls; the falls are bad because they easily injure the head

**Interviewer**: How would you comment on the current available guidelines concerning adherence to anti-seizure medications?

**Respondent**: Good guidelines are available, however healthcare providers only consider what we tell them when we come to the clinic of which some may not be correct, they should think of that, true, they should think of that

**Interviewer**: that is the end, thank you for participating
