# Supplementary material for: Adherence to anti-seizure medications among persons living with epilepsy attending a community- based epilepsy clinic in Buikwe and Mukono Districts-Uganda: A mixed methods study
Source: PLoS One. 2026 Aug 21;21(8):e0356494. doi: 10.1371/journal.pone.0356494 (PMC13496452; doi:10.1371/journal.pone.0356494)
Supplement: Peer review history

# Peer review: Decision Letter 0

**DOI:** 10.1371/journal.pone.0356494.r001

**Authors:** Marwan Al-Nimer

26 Oct 2025

PONE-D-25-46258

Adherence to anti-seizure medications among patients with epilepsy attending a community- based epilepsy clinic in Buikwe and Mukono districts-Uganda: a mixed methods study.

PLOS ONE

Dear Dr. kibirige,

Thank you for submitting your manuscript to PLOS ONE. After careful consideration, we have decided that your manuscript does not meet our criteria for publication and must therefore be rejected.

Specifically:

==============================

**ACADEMIC EDITOR: Declined**

==============================

I am sorry that we cannot be more positive on this occasion, but hope that you appreciate the reasons for this decision.

Kind regards,

Marwan Salih Al-Nimer, MD, PhD

Academic Editor

PLOS ONE

Additional Editor Comments :

There are some remarks about this manuscript

1: There are typing errors and abbreviations without explanation

2: More details need about the study design

3: A wide range of age will bias the results, particularly with studies on the epilepsy and medication adherence

4. The sample size was not calculated

5: The validity of the questionnaire is not clear and not reported

6: The Cronbach's alpha is not calculated

7: The level of significance 0.2?????? whether one or two-tailed?

8: Tables are not well prepared; the headings and subheadings of the variables were in separated rows.

9: The data of Morisky-8 and its analysis are not reported

10: The results of the Table 3 are not informative

11: Statistical analyses are inappropriate

12: References are not like the Style of the Journal

[Note: HTML markup is below. Please do not edit.]

Reviewers' comments:

Reviewer's Responses to Questions

**Comments to the Author**

1. Is the manuscript technically sound, and do the data support the conclusions?

Reviewer #1: Yes

**********

2. Has the statistical analysis been performed appropriately and rigorously?

Reviewer #1: Yes

**********

3. Have the authors made all data underlying the findings in their manuscript fully available?

The PLOS Data policy

Reviewer #1: Yes

**********

4. Is the manuscript presented in an intelligible fashion and written in standard English?

Reviewer #1: Yes

**********

Reviewer #1: When reviewing the manuscript, one of the first aspects that requires attention is the consistency in formatting and reference style, particularly the placement of citations. It is important to clearly decide whether there should be a space before the references and to ensure that this is applied uniformly throughout the entire text. Consistent referencing improves readability and professional presentation, which is particularly relevant in a journal like PLOS ONE.

With respect to the methodological aspects, I would appreciate further clarification regarding the recruitment process of the study participants. Specifically, how exactly were patients approached and invited to participate in the study? Were they directly asked by a clinician or research assistant if they wished to join the study, or was another strategy employed? It would be valuable to know whether data were collected on patients who declined participation. If such information exists, it could provide an important perspective on potential recruitment bias. Moreover, describing the clinical and socio-economic features of those who did not agree to participate would offer useful insights into the generalizability of the study findings.

Another important element relates to the language and validation of the questionnaire. In which language was the Morisky Medication Adherence Scale administered? Was this version formally validated for use in this population, and if so, could the authors provide the reference to the validation study or describe the adaptation process? If not, it may be important to acknowledge this as a potential limitation.

Regarding the section on “Characteristics of the study respondents”, I would recommend providing comparative data from the general population or from available regional statistics. Such a comparison would allow the reader to better understand how representative or distinctive this cohort is in relation to the broader population. It would also strengthen the epidemiological interpretation of the findings.

In the section titled “Characteristics of respondents for the qualitative study”, there appear to be several typographical and spacing errors, particularly around punctuation marks. A careful language and formatting review of this section is advised to ensure clarity and precision.

The finding of a surprisingly high adherence rate in this cohort compared to other studies conducted in similar resource settings, as well as in some high-income countries, deserves deeper reflection and contextualization. One possible explanation may be the younger age distribution of the sample, which could be associated with stronger family involvement, including support from parents or siblings. It may also be relevant to consider the role of community support systems, health worker–patient relationships, or programmatic factors such as medication availability and accessibility.

In addition to these points, I would suggest the authors consider a few further clarifications and expansions:

It would be beneficial to provide more information on whether adherence was cross-checked through any objective measure (e.g., pill counts, pharmacy refill records, or serum levels) or whether it relied solely on self-report. Even if not available, a brief reflection on this limitation would be appropriate.

The authors may wish to describe how missing data were handled during analysis and whether there was any imputation or sensitivity testing.

Given that long waiting times and treatment adequacy were identified as factors associated with adherence, it could be helpful to expand the discussion on structural or health system factors that might influence adherence in this setting.

Since the study uses a mixed-methods approach, a clearer description of how the qualitative and quantitative findings were integrated could strengthen the methodological rigor of the paper.

Overall, the manuscript addresses an important public health issue and provides valuable local data. With improved clarity regarding methods, formatting consistency, and expanded interpretation of findings, it has the potential to make a strong contribution to the literature on adherence to anti-seizure medications in low-resource settings.

**********

what does this mean?). If published, this will include your full peer review and any attached files.

If you choose “no”, your identity will remain anonymous but your review may still be made public.

**Do you want your identity to be public for this peer review?**  For information about this choice, including consent withdrawal, please see our Privacy Policy

Reviewer #1: **Yes:** Felix Benninger

**********

[NOTE: If reviewer comments were submitted as an attachment file, they will be attached to this email and accessible via the submission site. Please log into your account, locate the manuscript record, and check for the action link "View Attachments". If this link does not appear, there are no attachment files.]

- - - - -

For journal use only: PONEDEC3

# Author comment: Author response to Decision Letter 1

**DOI:** 10.1371/journal.pone.0356494.r002

25 Dec 2025

1. Reviewer #1: When reviewing the manuscript, one of the first aspects that requires attention is the consistency in formatting and reference style, particularly the placement of citations. It is important to clearly decide whether there should be a space before the references and to ensure that this is applied uniformly throughout the entire text. Consistent referencing improves readability and professional presentation, which is particularly relevant in a journal like PLOS ONE.

Formatting and referencing style has been corrected

2. With respect to the methodological aspects, I would appreciate further clarification regarding the recruitment process of the study participants. Specifically, how exactly were patients approached and invited to participate in the study? Were they directly asked by a clinician or research assistant if they wished to join the study, or was another strategy employed? It would be valuable to know whether data were collected on patients who declined participation. If such information exists, it could provide an important perspective on potential recruitment bias. Moreover, describing the clinical and socio-economic features of those who did not agree to participate would offer useful insights into the Generalizability of the study findings.

This has been addressed as follows;

• How exactly where patients approached and invited to participate in the study?

Participants were approached through the mental health care workers and the responsible community health workers who introduced the research team to the study respondents.

• It would be valuable to know whether data were collected on patients who declined participation

During data collection, 2 respondents who were; a male and a female aged 30 and 50 years respectively from urban areas, and attending to Mukono General Hospital mental health clinic declined participation in the study.. Data was not collected from these respondents.

3. Another important element relates to the language and validation of the questionnaire. In which language was the Morisky Medication Adherence Scale administered? Was this version formally validated for use in this population, and if so, could the authors provide the reference to the validation study or describe the adaptation process? If not, it may be important to acknowledge this as a potential limitation

Language the tool was administered

The tool was administered in Luganda, the language used in Buikwe and Mukono districts. This has been stated as follows: Two translators independently translated the English version of the MMAS-8 to Luganda, the dominant widely used language in Buikwe and Mukono districts, Uganda. A third translator performed reverse translation from Luganda to English. The original and back translated versions were compared and inconsistencies were resolved. A pilot test was performed in 10 patients with epilepsy and the interpretation of the tool was consistent. The patients who participated in this face- validity did not participate in this study.

• Validation of the MMAS-8 in this population

The MMAS-8 has been applied in studies measuring ASMs in Uganda but its specific validity for this use is derived from its general validation for chronic diseases. For hypertensive patients, the factor analysis of construct validity of the scale has been previously proven to be good with overall Kaiser’s measure of sampling adequacy for residuals of 0.72, and moderate internal consistency (Cronbach α=0.65). The MMAS-8 is one of the most widely used tools to measure medication adherence and has been validated in a wide range of population groups. References have been added to support this statement.

The MMAS-8 has not been previously validated among epilepsy patients in Uganda. However this tool has been validated among antihypertensive patients attending outpatient department in Uganda

This has also been reported as a limitation for this study

4. Regarding the section on “Characteristics of the study respondents”, I would recommend providing comparative data from the general population or from available regional statistics. Such a comparison would allow the reader to better understand how representative or distinctive this cohort is in relation to the broader population. It would also strengthen the epidemiological interpretation of the findings.

A statement on comparative data has been added in the introduction section as follows;

The national wide crude prevalence of epilepsy in Uganda is 16.9 per 1000 persons with an age- and sex- prevalence rates of 17.8 per 1000 among all individuals from one month to 108 years

5. In the section titled “Characteristics of respondents for the qualitative study”, there appear to be several typographical and spacing errors, particularly around punctuation marks. A careful language and formatting review of this section is advised to ensure clarity and precision.

Punctuation and language review has been done for this section

6. The finding of a surprisingly high adherence rate in this cohort compared to other studies conducted in similar resource settings, as well as in some high-income countries, deserves deeper reflection and contextualization. One possible explanation may be the younger age distribution of the sample, which could be associated with stronger family involvement, including support from parents or siblings. It may also be relevant to consider the role of community support systems, health worker–patient relationships, or programmatic factors such as medication availability and accessibility.

In addition to these points, I would suggest the authors consider a few further clarifications and expansions

This has been expanded as follows;

The higher prevalence of adherence to ASMs observed in this study compared to findings from several other studies may have been attributed to contextual and behavioral factors unique to the study population. Participants in this study setting may have high perceptions about the seriousness of epilepsy and benefits of consistent ASMs use, reinforced by strong family and community support, additionally close patient provider interaction and regular follow-up at the facilities may have enhanced patient’s understanding of adherence importance.

The consistent availability of ASMs at the facilities and the relatively easy access to treatment may have encouraged patients to adhere more to the prescribed regimens. When patients can readily access their medications without frequent stock outs, long distance travel or high costs, they are more likely to utilize their medications well, in contrast in some developed countries, despite advanced health care systems, factors such as complex prescription procedures and high treatment costs may limit consistent access and lead to lower adherence.

The higher prevalence of adherence to ASMs observed in this study may also be attributed to the young age distribution of the study participants. Younger individuals often receive stronger support from their siblings, demonstrate greater adaptability to treatment routines, and demonstrate better cognitive abilities to remember medication schedules

7. It would be beneficial to provide more information on whether adherence was cross-checked through any objective measure (e.g., pill counts, pharmacy refill records, or serum levels) or whether it relied solely on self-report. Even if not available, a brief reflection on this limitation would be appropriate.

This has been addressed as a limitation of the study and stated as follows;

Adherence to anti-seizure medications was solely self- reported which might have led to social desirability bias. We recommended a further study focusing on using blood levels other than self-report in determining adherence to ASMs would produce more quality results

8. The authors may wish to describe how missing data were handled during analysis and whether there was any imputation or sensitivity testing

This has been added in the manuscript and stated as follows;

Data were sorted, edited and entered into excel sheet. Data cleaning was then done to ensure consistence and completeness.

Additionally, research assistants were adequately and regularly supervised. The supervision entailed checking on the completeness and accuracy of the completed data collection forms.

9. Given that long waiting times and treatment adequacy were identified as factors associated with adherence, it could be helpful to expand the discussion on structural or health system factors that might influence adherence in this setting.

These have been addresses as follows;

Longer waiting time often reflects systematic inefficiencies such as staff shortages, inadequate patient flow management and overcrowded clinics. These challenges can discourage patients from attending to the clinics or refilling prescriptions, ultimately lowering adherence to ASMs among people with epilepsy.

Structural and health system related factors can influence the quality of services provided to patients. There is limited drug supply, poor procurement procedures, and inconsistent distribution of drugs which contribute to patients receiving inadequate dosages which can undermine treatment effectiveness and reduce motivation to adherence

10. Since the study uses a mixed-methods approach, a clearer description of how the qualitative and quantitative findings were integrated could strengthen the methodological rigor of the paper.

A section on integrating the research findings has been added and described as follows;

Quantitatively 66.5% of participants adhered to ASMs, with higher adherence observed among those earning higher monthly incomes, while low adherence was associated with inadequate dosages, longer waiting time and being aged 30 to 34 years. Qualitative findings complimented these findings by revealing that adherence was influenced by fear of poor consequences of epilepsy, individual motivation, and social support, whereas financial burdens, misconception about medications, and lack of awareness about ASMs effectiveness hindered consistent use. These findings suggest that although economic burden and health system related factors play a significant role in determining adherence levels, patient’s perceptions, social contexts, and motivation strongly shape ASMs adherence in daily life.

Additional Editor Comments:

There are some remarks about this manuscript

1: There are typing errors and abbreviations without explanation

We have corrected the typing errors and revised the abbreviations

2: More details need about the study design

More details have been added on the study design as follows;

Study design and population

The study was a cross-sectional design conducted both in the community and at the hospital, employing quantitative and qualitative methods. The study adhered to the Strengthening the Reporting of Observational Studies in Epidemiology (STROBE) guidelines. The study population consisted of Persons living with epilepsy 15 years and above attending regularly at the community-based epilepsy clinic in Buikwe District, and Mukono General Hospital, Mukono District. The study was carried from 05th April to 20th July 2024. Participants were approached through the mental health care workers, and the community health workers who introduced the research team to the study respondents.

3: A wide range of age will bias the results, particularly with studies on the epilepsy and medication adherence

We have explained this as follows;

First, we have re-categorized age from a range of 10 to 5 years to eliminate bias of the results. Secondly, Epilepsy being a rare condition in our settings, we recruited participants from 15 years and above in order to meet the sample size. Thirdly, the number of patients aged 15 to 19 years is reportedly high in these settings. Recruiting patients aged 15 and above has also been considered as a limitation of the study

4. The sample size was not calculated

We have indicated the details of how the sample size was calculated as follows:

For quantitative data, a sample size of 277 was determined using the modified formula for cross-sectional studies by Kish- Leslie 1965

N = z2p (1-p)/d2

Where N is the sample size

d = level of precision/ error, a precision of 5%

z = standard normal deviation corresponding to a 95% confidence interval, 1.96

p = the assumed probability of patients with adherence to anti-seizure medications; p=0.209

N= (1.962 x 0.209x0.791)

0.052

= 254

To account for potential non response, an additional 10% were included

(254*0.1) + 254

= 279

During data collection, 2 respondents who were; a male and a female aged 30 and 50 years respectively from urban areas, and attending to Mukono General Hospital mental health clinic declined participation in the study. Therefore; 277 study participants were enrolled for the quantitative part of the study

5: The validity of the questionnaire is not clear and not reported

The validity of the questionnaire has been explained and elaborated as follows;

The MMAS-8 has been applied in studies measuring ASMs in Uganda but its specific validity for this use is derived from its general validation for chronic diseases. For hypertensive patients, the factor analysis of construct validity of the scale has been previously proven to be good with overall Kaiser’s measure of sampling adequacy for residuals of 0.72, and moderate internal consistency (Cronbach α=0.65). The MMAS-8 is one of the most widely used tools to measure medication adherence and has been validated in a wide range of population groups. References have been added to support this statement.

The MMAS-8 has not been previously validated among epilepsy patients in Uganda. However this tool has been validated among antihypertensive patients attending outpatient department in Uganda

This has also been reported as a limitation for this study

6: The Cronbach's alpha is not calculated

Cronbach’s alpha has been calculated as indicated in the equation below

Calculating the Chronbach’s Alpha

α=(K)/(K-1)*(Sy2-∑Si2)/Sy2)

Where;

α= Chrombach’s alpha

K=number of items in the scale

Si=sum of item scores for each item

S=sum of total scores for all the items

α= (8/8-1)*(22162-14772)/22152

α=0.64

7: The level of significance 0.2?????? whether one or two-tailed?s

This has been corrected and stated as follows

A p-value threshold of 0.05 was used to assess for statistically significant associations

8: Tables are not well prepared; the headings and subheadings of the variables were in separated rows.

The headings and subheadings of the variables have been put in the same rows

9: The data of Morisky-8 and its analysis are not reported

Data of the MMAS-8 and how it was analyzed has been included

10: The results of the Table 3 are not informative

To make the contents of table 3 more formative, we have included text information instead of the table

11: Statistical analyses are inappropriate

Statistical analysis has been described further for clarity

12: References are not like the Style of the Journal

References have been changed to the style of the journal

Attachment Submitted filename: response to comments.docx

# Peer review: Decision Letter 1

**DOI:** 10.1371/journal.pone.0356494.r003

**Authors:** Hiroki Annaka

1 Jun 2026

Dear Dr. kibirige,

Thank you for submitting your manuscript to PLOS ONE. After careful consideration, we feel that it has merit but does not fully meet PLOS ONE’s publication criteria as it currently stands. Therefore, we invite you to submit a revised version of the manuscript that addresses the points raised during the review process.

==============================

==============================

Please submit your revised manuscript by Jul 16 2026 11:59PM. If you will need more time than this to complete your revisions, please reply to this message or contact the journal office at plosone@plos.org. When you're ready to submit your revision, log on to https://www.editorialmanager.com/pone/ and select the 'Submissions Needing Revision' folder to locate your manuscript file.

- A letter that responds to each point raised by the academic editor and reviewer(s). You should upload this letter as a separate file labeled 'Response to Reviewers'.
- A marked-up copy of your manuscript that highlights changes made to the original version. You should upload this as a separate file labeled 'Revised Manuscript with Track Changes'.
- An unmarked version of your revised paper without tracked changes. You should upload this as a separate file labeled 'Manuscript'.

If you would like to make changes to your financial disclosure, please include your updated statement in your cover letter. Guidelines for resubmitting your figure files are available below the reviewer comments at the end of this letter.

If applicable, we recommend that you deposit your laboratory protocols in protocols.io to enhance the reproducibility of your results. Protocols.io assigns your protocol its own identifier (DOI) so that it can be cited independently in the future. For instructions see: https://journals.plos.org/plosone/s/submission-guidelines#loc-laboratory-protocols. Additionally, PLOS ONE offers an option for publishing peer-reviewed Lab Protocol articles, which describe protocols hosted on protocols.io. Read more information on sharing protocols at https://plos.org/protocols?utm_medium=editorial-email&utm_source=authorletters&utm_campaign=protocols.

As the corresponding author, your ORCID iD is verified in the submission system and will appear in the published article. PLOS supports the use of ORCID, and we encourage all coauthors to register for an ORCID iD and use it as well. Please encourage your coauthors to verify their ORCID iD within the submission system before final acceptance, as unverified ORCID iDs will not appear in the published article. *Only*  the individual author can complete the verification step; PLOS staff *cannot*  verify ORCID iDs on behalf of authors.

We look forward to receiving your revised manuscript.

Kind regards,

Hiroki Annaka

Academic Editor

PLOS One

Journal Requirements:

1. When submitting your revision, we need you to address these additional requirements.

Please ensure that your manuscript meets PLOS ONE’s style requirements, including those for file

naming. The PLOS ONE style templates can be found at

https://journals.plos.org/plosone/s/file?id=wjVg/PLOSOne_formatting_sample_main_body.pdf and

https://journals.plos.org/plosone/s/file?id=ba62/PLOSOne_formatting_sample_title_authors_affiliation

s.pdf

2. Please include a complete copy of PLOS’ questionnaire on inclusivity in global research in your

revised manuscript. Our policy for research in this area aims to improve transparency in the

reporting of research performed outside of researchers’ own country or community. The policy

applies to researchers who have travelled to a different country to conduct research, research

with Indigenous populations or their lands, and research on cultural artefacts. The questionnaire

can also be requested at the journal’s discretion for any other submissions, even if these

conditions are not met. Please find more information on the policy and a link to download a

blank copy of the questionnaire here: https://journals.plos.org/plosone/s/best-practices-in-

research-reporting. Please upload a completed version of your questionnaire as Supporting

Information when you resubmit your manuscript.

3. We note that the grant information you provided in the ‘Funding Information’ and ‘Financial

Disclosure’ sections do not match.

When you resubmit, please ensure that you provide the correct grant numbers for the awards you

received for your study in the ‘Funding Information’ section.

4. Thank you for stating in your Funding Statement:

Research reported in this publication was supported by the National Institute Of Neurological Disorders

And Stroke of the National Institutes of Health under Award Number R01NS118544. The content is

solely the responsibility of the authors and does not necessarily represent the official views of the

National Institutes of Health

Please provide an amended statement that declares *all* the funding or sources of support (whether

external or internal to your organization) received during this study, as detailed online in our guide for

authors at http://journals.plos.org/plosone/s/submit-now. Please also include the statement “There

was no additional external funding received for this study.” in your updated Funding Statement.

Please include your amended Funding Statement within your cover letter. We will change the online

submission form on your behalf.

5. Thank you for stating the following financial disclosure:

Research reported in this publication was supported by the National Institute Of Neurological Disorders

And Stroke of the National Institutes of Health under Award Number R01NS118544. The content is

solely the responsibility of the authors and does not necessarily represent the official views of the

National Institutes of Health

Please state what role the funders took in the study. If the funders had no role, please state: &quot;The

funders had no role in study design, data collection and analysis, decision to publish, or preparation of

the manuscript.&quot;

If this statement is not correct you must amend it as needed.

Please include this amended Role of Funder statement in your cover letter; we will change the online

submission form on your behalf.

6. We note that your Data Availability Statement is currently as follows: All relevant data are

within the manuscript and its Supporting Information files.

Please confirm at this time whether or not your submission contains all raw data required to replicate

the results of your study. Authors must share the “minimal data set” for their submission. PLOS defines

the minimal data set to consist of the data required to replicate all study findings reported in the article,

as well as related metadata and methods (https://journals.plos.org/plosone/s/data-availability#loc-

minimal-data-set-definition).

For example, authors should submit the following data:

- The values behind the means, standard deviations and other measures reported;

- The values used to build graphs;

- The points extracted from images for analysis.

Authors do not need to submit their entire data set if only a portion of the data was used in the

reported study.

If your submission does not contain these data, please either upload them as Supporting Information

files or deposit them to a stable, public repository and provide us with the relevant URLs, DOIs, or

accession numbers. For a list of recommended repositories, please see

https://journals.plos.org/plosone/s/recommended-repositories.

If there are ethical or legal restrictions on sharing a de-identified data set, please explain them in detail

(e.g., data contain potentially sensitive information, data are owned by a third-party organization, etc.)

and who has imposed them (e.g., an ethics committee). Please also provide contact information for a

data access committee, ethics committee, or other institutional body to which data requests may be

sent. If data are owned by a third party, please indicate how others may request data access.

7. PLOS requires an ORCID iD for the corresponding author in Editorial Manager on papers

submitted after December 6th, 2016. Please ensure that you have an ORCID iD and that it is

validated in Editorial Manager. To do this, go to ‘Update my Information’ (in the upper left-hand

corner of the main menu), and click on the Fetch/Validate link next to the ORCID field. This will

take you to the ORCID site and allow you to create a new iD or authenticate a pre-existing iD in

Editorial Manager.

8. Please amend either the title on the online submission form (via Edit Submission) or the title in

the manuscript so that they are identical.

9. Your ethics statement should only appear in the Methods section of your manuscript. If your

ethics statement is written in any section besides the Methods, please move it to the Methods

section and delete it from any other section. Please ensure that your ethics statement is

included in your manuscript, as the ethics statement entered into the online submission form

will not be published alongside your manuscript.

If the reviewer comments include a recommendation to cite specific previously published works, please review and evaluate these publications to determine whether they are relevant and should be cited. There is no requirement to cite these works unless the editor has indicated otherwise.

Additional Editor Comments (if provided):

[Note: HTML markup is below. Please do not edit.]

Reviewers' comments:

Reviewer's Responses to Questions

**Comments to the Author**

Reviewer #2: (No Response)

Reviewer #3: (No Response)

**********

2. Is the manuscript technically sound, and do the data support the conclusions?

Reviewer #2: Yes

Reviewer #3: Partly

**********

3. Has the statistical analysis been performed appropriately and rigorously?

Reviewer #2: Yes

Reviewer #3: I Don't Know

**********

4. Have the authors made all data underlying the findings in their manuscript fully available?

The PLOS Data policy

Reviewer #2: Yes

Reviewer #3: Yes

**********

5. Is the manuscript presented in an intelligible fashion and written in standard English?

Reviewer #2: Yes

Reviewer #3: Yes

**********

Reviewer #2: 1) avoid using abbreviation in the Abstract, line 48.

2) The first sentence in the introduction section "Epilepsy is a chronic neurological disorder that affects 50 million people worldwide of which 80% of them reside in developing countries where 64 per 100,000 inhabitants are affected per year" need slight grammatical check, authors need to pay more attention to punctuation marks throughout the manuscript; which would improve the readability of the text.

3) The sentence "Hospital records show that 200 and 100 patients 15 years and above receive treatment from these sites respectively", line 123, is not clear.

4) Please provide adequate explanation for how the 20 patients were selected out of the 277 patients to participate in the qualitative interviews, this is required to guard against potential bias.

5) The paragraph starting in line 126 is repeated in line 150, this should be corrected.

6) English should be revised throughout the manuscript for minor grammatical errors.

Reviewer #3: Thank you for the opportunity to review this manuscript on an important topic on epilepsy care in Uganda. Medication adherence is a key component to effective self-management in epilepsy and this study to understand the prevalence and factors influencing adherence in both a general hospital and community-based clinic setting is a valuable contribution to the field. The authors appear to have satisfactorily addressed the prior reviewers' comments.

A few addressable concerns temper my enthusiasm for immediate acceptance of this version of the paper.

1) The calculation of the % adherence in this cohort appears (from page 16 - 17 table and methods) to be derived from the number of answers to individual MMAS questions that fall into the adherence vs low adherence categories, rather than based on the number of individuals whose total MMAS score was above or below the adherent vs moderate/low adherent cut off. I would have expected to see a % that reflects the number of people who are classified adherent out of the total number of people in the cohort. Later in the paper (p 30) the % is referred to as "66.5% of participants adhered to ASMs."

2) Is it appropriate to lump the participants from the two locations into a single cohort? There were no descriptive comparisons made between the participants enrolled from the community-based clinic and the general hospital that argued for their being a reasonably homogeneous group. If there are key differences in age, income level, distance from the site, etc. between participants at the two locations, it may have been more interesting and informative to compare whether there are differences between factors influencing adherence that occur at the two sites.

3) Religious affiliation is reported and mentioned as a factor with Pentacostal prevalence noted in particular, but there is no further explanation or discussion about religious affiliation's relationship to the results.

4) Although it may have been beyond the scope of this study, it would be good to mention whether the investigators identified ways to attempt to address any modifiable barriers to adherence that they uncovered or assist participants with creating adherence plans that could help improve adherence. While things like inconsistent medication supply at the site may not be something that can be addressed at an individual level, things like forgetting, unwanted side effects, or misunderstandings about how the medication works could potentially addressed for individuals.

Overall, the methods are sound and the conclusions are supported by the data. Appropriate limitations are noted. I recommend the authors make minor revisions to address these items and resubmit the paper for subsequent publication in PLOS.

**********

what does this mean?). If published, this will include your full peer review and any attached files.

If you choose “no”, your identity will remain anonymous but your review may still be made public.

**Do you want your identity to be public for this peer review?**  For information about this choice, including consent withdrawal, please see our Privacy Policy

Reviewer #2: **Yes:** Aseel Adil Ahmed Abdelhafiz

Reviewer #3: **Yes:** Brandy E. Fureman, PhD, FAES

**********

[NOTE: If reviewer comments were submitted as an attachment file, they will be attached to this email and accessible via the submission site. Please log into your account, locate the manuscript record, and check for the action link "View Attachments". If this link does not appear, there are no attachment files.]

To ensure your figures meet our technical requirements, please review our figure guidelines: https://journals.plos.org/plosone/s/figures

You may also use PLOS’s free figure tool, NAAS, to help you prepare publication quality figures: https://journals.plos.org/plosone/s/figures#loc-tools-for-figure-preparation.

NAAS will assess whether your figures meet our technical requirements by comparing each figure against our figure specifications.

# Author comment: Author response to Decision Letter 2

**DOI:** 10.1371/journal.pone.0356494.r004

3 Jul 2026

7/2/2026

The Editor,

PLOS One,

On behalf of the authors, I thank you for a quick review of our manuscript. We have responded to the comments raised as indicated in italics below. We have uploaded a clean manuscript in the submission system.

COMMENTS FROM THE EDITOR

The discussion of the results needs to be further developed.

The discussion section has been further developed

Journal Requirements:

1. When submitting your revision, we need you to address these additional requirements.

Please ensure that your manuscript meets PLOS ONE's style requirements, including those for filenaming. The PLOS ONE style templates can be found at https://journals.plos.org/plosone/s/file?id=wjVg/PLOSOne_formatting_sample_main_body.pdf and https://journals.plos.org/plosone/s/file?id=ba62/PLOSOne_formatting_sample_title_authors_affiliations.pdf

We have improved the manuscript to meet the journal requirements

2. Please include a complete copy of PLOS’ questionnaire on inclusivity in global research in your

revised manuscript. Our policy for research in this area aims to improve transparency in the

reporting of research performed outside of researchers’ own country or community. The policy

applies to researchers who have travelled to a different country to conduct research, research

with Indigenous populations or their lands, and research on cultural artefacts. The questionnaire

can also be requested at the journal’s discretion for any other submissions, even if these

conditions are not met. Please find more information on the policy and a link to download a

blank copy of the questionnaire here: https://journals.plos.org/plosone/s/best-practices-in-

research-reporting. Please upload a completed version of your questionnaire as Supporting

Information when you resubmit your manuscript.

A completed copy of PLOS ONE questionnaire has been included in the supporting information labeled X1 Checklist

3. We note that the grant information you provided in the ‘Funding Information’ and ‘Financial

Disclosure’ sections do not match.When you resubmit, please ensure that you provide the correct grant numbers for the awards youreceived for your study in the ‘Funding Information’ section.

The funding information and financial disclosure statement have been improved including the correct grant numbers for the awards.

Funding

Research reported in this publication was supported by the National Institute of Neurological Disorders and Stroke of the National Institutes of Health under Award Number 1D43NS118560-01. The content is solely the responsibility of the authors and does not necessarily represent the official views of the National Institutes of Health.

4. Thank you for stating in your Funding Statement:

We apologize for this mishap; the funding statement has been revised to indicate the correct funding number.

5. Thank you for stating the following financial disclosure.

Please include this amended Role of Funder statement in your cover letter; we will change the online submission form on your behalf.

We apologize for this mishap; the funding statement has been revised to indicate the correct funding number.

6. We note that your Data Availability Statement is currently as follows: All relevant data are

within the manuscript and its Supporting Information files.

Please confirm at this time whether or not your submission contains all raw data required to replicate the results of your study. Authors must share the “minimal data set” for their submission. PLOS defines the minimal data set to consist of the data required to replicate all study findings reported in the article, as well as related metadata and methods https://journals.plos.org/plosone/s/data-availability#loc-minimal-data-set-definition).

For example, authors should submit the following data:

The values behind the means, standard deviations and other measures reported;

The values used to build graphs;

The points extracted from images for analysis.

Authors do not need to submit their entire data set if only a portion of the data was used in the

reported study.

If your submission does not contain these data, please either upload them as Supporting Information files or deposit them to a stable, public repository and provide us with the relevant URLs, DOIs, or accession numbers. For a list of recommended repositories, please see

https://journals.plos.org/plosone/s/recommended-repositories.

If there are ethical or legal restrictions on sharing a de-identified data set, please explain them in detail (e.g., data contain potentially sensitive information, data are owned by a third-party organization, etc.) and who has imposed them (e.g., an ethics committee). Please also provide contact information for a data access committee, ethics committee, or other institutional body to which data requests may be sent. If data are owned by a third party, please indicate how others may request data access.

All relevant data required to replicate the results of this study have been provided in the supporting information files labeled S2_ quantitative data and S3_qualitative data

7. PLOS requires an ORCID iD for the corresponding author in Editorial Manager on papers

submitted after December 6th, 2016. Please ensure that you have an ORCID iD and that it is

validated in Editorial Manager. To do this, go to ‘Update my Information’ (in the upper left-hand

corner of the main menu), and click on the Fetch/Validate link next to the ORCID field. This will

take you to the ORCID site and allow you to create a new iD or authenticate a pre-existing iD in

Editorial Manager.

The corresponding author has created an ORCID iD (0009-0006-5563-0773)

8. Please amend either the title on the online submission form (via Edit Submission) or the title inthe manuscript so that they are identical.

The title has been amended as “Adherence to anti-seizure medications among persons living with epilepsy attending a community- based epilepsy clinic in Buikwe and Mukono Districts-Uganda: a mixed methods study”

9. Your ethics statement should only appear in the Methods section of your manuscript. If your

ethics statement is written in any section besides the Methods, please move it to the Methods

section and delete it from any other section. Please ensure that your ethics statement is

included in your manuscript, as the ethics statement entered into the online submission form

will not be published alongside your manuscript.

The ethics statement has been provided on page 15, lines 309 – 318.

COMMENTS FROM THE REVIEWERS

Reviewer #2:

1) Avoid using abbreviation in the abstract, line 48.

The abbreviation has been rewritten in full.

2) The first sentence in the introduction section "Epilepsy is a chronic neurological disorder that affects 50 million people worldwide of which 80% of them reside in developing countries where 64 per 100,000 inhabitants are affected per year" need slight grammatical check, authors need to pay more attention to punctuation marks throughout the manuscript; which would improve the readability of the text.

This has been rewritten and improved grammatically.

3)The sentence "Hospital records show that 200 and 100 patients 15 years and above receive treatment from these sites respectively", line 123, is not clear.

This has been rewritten to improve the clarity, see lines 144 -148, Page 7.

4) Please provide adequate explanation for how the 20 patients were selected out of the 277 patients to participate in the qualitative interviews, this is required to guard against potential bias.

This has been rewritten see lines 150 – 153, page 8.

5) The paragraph starting in line 126 is repeated in line 150, this should be corrected.

This has been corrected

6) English should be revised throughout the manuscript for minor grammatical errors.

Language and minor grammatical errors have been revised throughout the manuscript.

Reviewer #3

A few addressable concerns temper my enthusiasm for immediate acceptance of this version of the paper.

1) The calculation of the % adherence in this cohort appears (from page 16 - 17 table and methods) to be derived from the number of answers to individual MMAS questions that fall into the adherence vs low adherence categories, rather than based on the number of individuals whose total MMAS score was above or below the adherent vs moderate/low adherent cut off. I would have expected to see a % that reflects the number of people who are classified adherent out of the total number of people in the cohort. Later in the paper (p 30) the % is referred to as "66.5% of participants adhered to ASMs."

This has been rewritten see lines 273- 277, page 14.

2) Is it appropriate to lump the participants from the two locations into a single cohort? There were no descriptive comparisons made between the participants enrolled from the community-based clinic and the general hospital that argued for their being a reasonably homogeneous group. If there are key differences in age, income level, distance from the site, etc. between participants at the two locations, it may have been more interesting and informative to compare whether there are differences between factors influencing adherence that occur at the two sites.

We have included a justification for pooling these two sites, see lines 107 – 116, page 6.

3) Religious affiliation is reported and mentioned as a factor with Pentecostal prevalence noted in particular, but there is no further explanation or discussion about religious affiliation's relationship to the results.

Religious affiliation was not associated with ASMs adherence, this has been stated see lines 304 – 305, page 16.

4) Although it may have been beyond the scope of this study, it would be good to mention whether the investigators identified ways to attempt to address any modifiable barriers to adherence that they uncovered or assist participants with creating adherence plans that could help improve adherence. While things like inconsistent medication supply at the site may not be something that can be addressed at an individual level, things like forgetting, unwanted side effects, or misunderstandings about how the medication works could potentially addressed for individuals.

We have included a statement in the discussion regarding this, see lines 575 – 576, page 29.

Sincerely,

Ronald Kibirige

# Peer review: Decision Letter 2

**DOI:** 10.1371/journal.pone.0356494.r005

**Authors:** Hiroki Annaka

4 Aug 2026

Adherence to anti-seizure medications among persons living with epilepsy attending a community- based epilepsy clinic in Buikwe and Mukono Districts-Uganda: a mixed methods study.

PONE-D-25-46258R2

Dear Dr. kibirige,

We’re pleased to inform you that your manuscript has been judged scientifically suitable for publication and will be formally accepted for publication once it meets all outstanding technical requirements.

Within one week, you’ll receive an e-mail detailing the required amendments. When these have been addressed, you’ll receive a formal acceptance letter and your manuscript will be scheduled for publication.

An invoice will be generated when your article is formally accepted. Please note, if your institution has a publishing partnership with PLOS and your article meets the relevant criteria, all or part of your publication costs will be covered. Please make sure your user information is up-to-date by logging into Editorial Manager at Editorial Manager® and clicking the ‘Update My Information' link at the top of the page. For questions related to billing, please contact billing support.

If your institution or institutions have a press office, please notify them about your upcoming paper to help maximize its impact. If they’ll be preparing press materials, please inform our press team as soon as possible -- no later than 48 hours after receiving the formal acceptance. Your manuscript will remain under strict press embargo until 2 pm Eastern Time on the date of publication. For more information, please contact onepress@plos.org.

Kind regards,

Hiroki Annaka

Academic Editor

PLOS One

Additional Editor Comments (optional):

Reviewers' comments:

Reviewer's Responses to Questions

**Comments to the Author**

Reviewer #3: All comments have been addressed

**********

2. Is the manuscript technically sound, and do the data support the conclusions?

Reviewer #3: Yes

**********

3. Has the statistical analysis been performed appropriately and rigorously?

Reviewer #3: Yes

**********

4. Have the authors made all data underlying the findings in their manuscript fully available?

The PLOS Data policy

Reviewer #3: Yes

**********

5. Is the manuscript presented in an intelligible fashion and written in standard English?

Reviewer #3: Yes

**********

Reviewer #3: The authors have addressed all my prior comments and I have no remaining issues with the manuscript. There are typographical errors in spacing throughout, but I assume those can be dealt with in proofs.

**********

what does this mean?). If published, this will include your full peer review and any attached files.

If you choose “no”, your identity will remain anonymous but your review may still be made public.

**Do you want your identity to be public for this peer review?**  For information about this choice, including consent withdrawal, please see our Privacy Policy

Reviewer #3: **Yes:** Brandy E. Fureman

**********

# Editor report: Acceptance letter

**DOI:** 10.1371/journal.pone.0356494.r006

**Authors:** Hiroki Annaka

PONE-D-25-46258R2

PLOS One

Dear Dr. Kibirige,

I'm pleased to inform you that your manuscript has been deemed suitable for publication in PLOS One. Congratulations! Your manuscript is now being handed over to our production team.

At this stage, our production department will prepare your paper for publication. This includes ensuring the following:

* All references, tables, and figures are properly cited

* All relevant supporting information is included in the manuscript submission,

* There are no issues that prevent the paper from being properly typeset

You will receive further instructions from the production team, including instructions on how to review your proof when it is ready. Please keep in mind that we are working through a large volume of accepted articles, so please give us a few days to review your paper and let you know the next and final steps.

Lastly, if your institution or institutions have a press office, please let them know about your upcoming paper now to help maximize its impact. If they'll be preparing press materials, please inform our press team within the next 48 hours. Your manuscript will remain under strict press embargo until 2 pm Eastern Time on the date of publication. For more information, please contact onepress@plos.org.

You will receive an invoice from PLOS for your publication fee after your manuscript has reached the completed accept phase. If you receive an email requesting payment before acceptance or for any other service, this may be a phishing scheme. Learn how to identify phishing emails and protect your accounts at https://explore.plos.org/phishing.

If we can help with anything else, please email us at customercare@plos.org.

Thank you for submitting your work to PLOS ONE and supporting open access.

Kind regards,

PLOS ONE Editorial Office Staff

on behalf of

Dr. Hiroki Annaka

Academic Editor

PLOS One
